# Person‐centred integrated care for people living with Parkinson's, Huntington's and Multiple Sclerosis: A systematic review

**DOI:** 10.1111/hex.13948

**Published:** 2023-12-27

**Authors:** Sandra Bartolomeu Pires, Dorit Kunkel, Christopher Kipps, Nick Goodwin, Mari C. Portillo

**Affiliations:** ^1^ NIHR Applied Research Collaboration Wessex, Southampton Science Park, Innovation Centre Southampton UK; ^2^ School of Health Sciences University of Southampton Southampton UK; ^3^ Department of Clinical and Experimental Sciences, Faculty of Medicine University of Southampton Southampton UK; ^4^ Wessex Neurological Centre University Hospital Southampton NHS Foundation Trust Southampton UK; ^5^ Central Coast Research Institute for Integrated Care, College of Health Medicine and Wellbeing University of Newcastle Newcastle New South Wales Australia

**Keywords:** Huntington's disease, integrated care, Multiple Sclerosis, multisectoral, Parkinson's disease, person‐centred outcomes

## Abstract

**Introduction:**

People living with long‐term neurological conditions (LTNCs) have complex needs that demand intensive care coordination between sectors. This review aimed to establish if integrated care improves outcomes for people, and what characterises successful interventions.

**Methods:**

A systematic review of the literature was undertaken evaluating multisectoral integrated care interventions in people living with Parkinson's disease (PD), Multiple Sclerosis (MS) and Huntington's disease (HD). Strength of evidence was rated for the different outcomes.

**Results:**

A total of 15 articles were included, reporting on 2095 patients and caregivers, finding that integrated care can improve people's access to resources and reduce patients' depression. UK studies indicated improvements in patients' quality of life, although the international literature was inconclusive. Few programmes considered caregivers' outcomes, reporting no difference or even worsening in depression, burden and quality of life. Overall, the evidence showed a mismatch between people's needs and outcomes measured, with significant outcomes (e.g., self‐management, continuity of care, care experience) lacking. Successful programmes were characterised by expert knowledge, multisectoral care coordination, care continuity and a person‐centred approach.

**Conclusions:**

The impact of integrated care programmes on people living with LTNCs is limited and inconclusive. For a more person‐centred approach, future studies need to assess integrated care from a service‐user perspective.

**Patient and Public Contribution:**

Thirty people living with LTNCs were involved in this review, through defining research questions, validating the importance of the project, and increasing the researchers' understanding on what matters to service users. A patient and public involvement subgroup of representatives with lived experience on PD, MS and HD identified the need for more person‐centred integrated care, with specific concerns over care fragmentation, care duplication and care continuity. This was key to data analysis and formulating the characteristics of successful and unsuccessful integrated care programmes from the perspective of service users. The discrepancy between service users' needs and the outcomes assessed in the literature point to user‐driven research as the solution to address what matters to patients and caregivers.

## 1 INTRODUCTION

Long‐term neurological conditions (LTNCs) pose a large and increasing burden globally in terms of disability, mortality and costs.^1^, ^2^, ^3^, ^4^ As the prevalence of LTNCs increases, governments face increasing demands for treatment, rehabilitation and support services.^1^ People living with LTNCs have complex needs that require multidimensional care.^5^, ^6^, ^7^, ^8^ Both motor and non‐motor features result in self‐management difficulty, increased dependence, and caregivers' burden. With more than 600 neurological conditions,^9^ it is crucial to understand the commonalities across conditions for a better integrated service response.

Evidence shows that people living with Parkinson's disease (PD), Multiple Sclerosis (MS) and Huntington's disease (HD) have common unmet needs that negatively impact on their experiences of care and care outcomes:

1. Access to care^10^, ^11^, ^12^, ^13^, ^14^, ^15^, ^16^, ^17^, ^18^, ^19^, ^20^, ^21^, ^22^, ^23^, ^24^, ^25^, ^26^
2. Care continuity and coordination between providers^10^, ^11^, ^12^, ^13^, ^14^, ^15^, ^18^, ^19^, ^20^, ^22^, ^23^, ^26^, ^27^, ^28^, ^29^
3. Collaboration between providers with a shared care plan^12^, ^19^, ^29^, ^30^
4. Personalized care and institutional flexibility^10^, ^12^, ^14^, ^24^, ^27^, ^31^
5. Financial, psychological and social support^12^, ^14^, ^15^, ^16^, ^18^, ^23^, ^30^, ^32^, ^33^, ^34^, ^35^, ^36^
6. Proactive care^12^, ^13^, ^14^
7. Community resources and support^13^, ^15^, ^16^, ^18^, ^23^, ^29^, ^31^, ^33^
8. Expert staff^14^, ^20^, ^23^, ^31^, ^36^ and,
9. Information adapted to the disease journey.^11^, ^12^, ^15^, ^17^, ^22^, ^24^, ^26^, ^36^

People living with rare neurological diseases, such as those impacted by HD, seem to face additional needs, as reported in the National Neurological Patient Experience Survey 2018/2019^37^ from more than 10,000 people; this resulted in a follow‐up report specifically looking at the needs of people living with rare neurological diseases^38^. Particularly looking at HD, their needs differ from PD and MS, due to its rare and hereditary nature, whose impact is stated in the literature as: isolation,^39^ lack of professional and public awareness^31^, ^36^, ^39^; limited resources (e.g., many long‐term facilities not accepting HD patients)^29^, ^31^, ^36^; and familial needs.^35^, ^39^ Some of these extended needs were explored by a recent survey where, even within a rare disease scope, people with HD and other choreas experienced higher difficulties in accessing care. Reasons for this were the small number of experts who usually work at public and private institutions, expensive consultations, long waiting lists and lack of knowledge amongst clinicians.^40^

These unmet needs demonstrate how fragmented care delivery undermines the capability to meet the complex care needs of people living with LTNCs. Policy suggests an integrated care response is needed.^41^, ^42^ The concept has evolved through time and taken several definitions.^43^, ^44^ This review adopted the definition from the World Health Organization (WHO)^41^, ^45^ because it aligns with the multisectoral care required in LTNCs—integrated care is delivered by a coordinated multidisciplinary team of providers working across settings and levels of care, through intersectoral and multisectoral actions. A multisectoral approach is understood as the collaboration between various stakeholder groups from: "macro (society structures at national or governmental levels), meso (middle groups of organizations like communities, voluntary sector or neighbourhoods) and micro (local individual level e.g., personal networks) societal levels of action”^46^ (p.8) to achieve policy, health and practice related outcomes.

Integrated care has shown improvements in other long‐term conditions like cancer, diabetes and cardiovascular diseases,^47^, ^48^, ^49^, ^50^, ^51^ increasing patient satisfaction, perceived quality of care, and access to care. Little is known though about its impact on people living with LTNCs. A 2010 rapid review^52^ pointed out that despite the growth in models of care being tested for people with LTNCs, the evidence base about the best models to adopt remained underdeveloped. The review highlighted that multidisciplinary work alongside clinical nurse specialists could improve care continuity, but patient‐focused outcomes were largely absent from the studies included. Indeed, the authors reported that fewer than half of the studies undertook any assessment from service users' perspective. With little comparative information available, the authors questioned if users' feedback reflected their gratitude for receiving any service, or rather if the model being tested was better than the standard of care.

The current review expands on the work by Parker et al.^52^ by taking a multisectoral approach to integrated care, a more developed and up‐to‐date concept than continuity of care by Freeman et al.^53^; second, it will specify a rare neurological condition (HD) aiming to build knowledge across prevalent and rare neurological conditions, for better services response; third, it will provide an update on the last two decades since Parker's search was conducted in 2006; lastly, it will employ a systematic and rigorous search with a service‐user perspective lenses to meet the gap on person‐centred outcomes.

To date, no systematic literature review has gathered knowledge across prevalent and rare LTNCs to understand the effect of integrated care programmes on this population. Therefore, this review aims to identify the key characteristics of successful integrated care programmes tested in people living with PD, MS and HD and their outcomes on patients and caregivers.

## 2 METHODS

### 2.1 Search strategy and selection criteria

The research team conducted a systematic review of the international evidence, examining the characteristics and impact of integrated care programmes in people living with PD, MS and HD. The protocol was registered on PROSPERO (number CRD42022314740).^54^ The review is reported in line with Preferred Reporting Items for Systematic Reviews and Meta‐Analyses guidelines^55^ (Supporting Information S1: Appendix 1).

CINAHL, Cochrane, Embase, PsycINFO, Medline, Web of Science and Google Scholar were searched, per Bramer's optimal database combination,^56^ using a comprehensive search strategy (Supporting Information S1: Appendix 2). Limits were used for articles published in English, German, Portuguese and Spanish languages, from 1 January 2000 (before, the literature focused on multidisciplinary work instead of multisectoral, trends evolved from whole systems working, integrated delivery networks and patient‐centred care^43^) to 30 September 2021.

Furthermore, other iterative searching techniques were employed, such as hand‐searching of issues published between 01 September 2020 and 13 May 2022 on *The Lancet Neurology*, *Movement Disorders*, *International Journal of Integrated Care* and *BMC Health Services Research*, to increase the sensitivity of the literature searches and minimize retrieval bias of the databases.^57^ Alerts were set on a variety of relevant journals using Zetoc and eligibility criteria was applied and regularly screened since 22 August 2020 (Supporting Information S1: Appendix 3). The systematic search was complemented with “snowball” methods (pursuing references of references and electronic citation tracking both forwards and backwards, up to the year 2000) and expert knowledge, strategies especially powerful for identifying high‐quality sources in obscure locations.^58^

The inclusion criteria applied were: (i) empirical studies exploring integrated care interventions for people diagnosed with PD, MS or HD and/or their informal caregivers; (ii) studies delivered by a multidisciplinary team working across different levels and sectors of care (iii) grey literature addressing this review aim.

The exclusion criteria were: (i) studies focused on disease management that omitted multilevel/multisectoral interventions; (ii) articles focused on other parkinsonian syndromes, other than idiopathic PD; (iii) commentaries, editorials, opinion pieces, conference abstracts. Literature reviews were excluded but articles within them were screened individually.

Retrieved citations were uploaded using Rayyan.^59^ Two independent researchers (S. B. P. and M. C. P.) screened the papers by titles and abstracts to assess their eligibility. Disagreements were taken to a third reviewer (D. K.) and discussed until consensus was reached. Eligible papers had the full‐text retrieved and analysed by two researchers (S. B. P. and D. K.); papers whose suitability could not be judged by title and abstract also had the full‐text retrieved. Any disagreements were taken blindly to the third researcher (M. C. P.) and discussed until consensus. Excluded papers and reasons for exclusion were recorded on Rayyan.

### 2.2 Data extraction and analysis

Data were extracted from full‐text papers meeting the inclusion and exclusion criteria using a template Excel spreadsheet inspired by Joanna Briggs Institute^60^ (Supporting Information S1: Appendix 4). Data for the study identifier, study design, context, population characteristics, type and details of the intervention, outcomes, study limitations and other comments were extracted. The template was tested^61^, ^62^ by extracting data from three articles^63^, ^64^, ^65^ (by S. B. P., M. C. P., D. K.). The testing focused on the clarity and completeness of each column heading on the template. The authors discussed confusing and/or incomplete instructions. This process identified data that was missing from the form, but also duplicated data, refining the data extraction template. Data for the remaining articles were extracted by one researcher (S. B. P.); in cases of uncertainty, a second researcher (M. C. P./D. K.) independently extracted data from the same article and results were compared and discussed until reaching consensus.

The main review outcomes of interest were: integrated care definition and characteristics, model of care, details of multidisciplinary, intersectoral/multisectoral interventions, roles involved and outcomes measured. Secondary outcomes were feasibility, obstacles and strategies to implementation. Data were analysed using tabulation and thematic analysis to compare the impact of interventions in patients, caregivers, and services/organisations. Three authors (S. B. P., D. K., M. C. P.) developed tables with relevant subheadings following the review questions, that is, author/year/country, intervention characteristics and effect, to understand the characteristics of successful and unsuccessful interventions.

Outcome measures were graded for strength, to report where there was greater or lesser strength (or certainty) in the evidence. This approach infers certainty based on two factors: the methodological quality of the individual studies and the plausibility of each study finding.^66^ It is important that any assessment of the strength of evidence considers the quality and volume of studies, but also considers consistency.^67^ This evaluation draws on work by Hoogendoorn,^67^ with principles from the GRADE and CERQUAL rating schemes,^66^, ^68^ and work from Baxter.^51^, ^69^ To evaluate the strength of the evidence comparator labels were used. The rating scale was as follows: ‘stronger evidence’ represented generally consistent findings (more than half) in multiple studies with a comparator group design; ‘weaker evidence’ represented generally consistent findings in one study with a comparator group design and several noncomparator studies, or multiple noncomparator studies; ‘very limited evidence’ represented an outcome reported by a single study; and finally, ‘inconsistent evidence’ represented an outcome where fewer than 75% of studies agreed on the direction of effect.

United Kingdom and international evidence with comparator and noncomparator studies were separately rated, and then an overall rating effect across study type was provided. Each outcome reported was recorded either by a plus ‘+’ meaning that the study reported an improvement for this outcome, by an equal sign ‘=’ meaning no significant change, or by a minus sign ‘−’ meaning a decline for this outcome. Following rating in individual studies, overall ratings were achieved across all evidence, grouping these in relation to patients, caregivers and resource use/system impact. Strength of evidence appraisal was undertaken by the research team (S. B. P., M. C. and D. K.) at a series of meetings to establish consensus.

The quality of the included studies was independently appraised by two authors (S. B. P., D. K.) applying the Critical Appraisal Skills Programme.^70^ For studies with comparative designs, the authors considered sources of potential bias based on the Cochrane Handbook.^71^ Disagreements were resolved by discussion and consensus with a third author (M. C. P.).

### 2.3 Patient and public involvement (PPI)

This systematic review was conducted with a PPI group composed of 30 adults living with a LTNC, either diagnosed, at risk (HD) or caring for someone impacted by a LTNC. Some PPI meetings were designed to meet with people with different disease experiences and discuss overlapping concerns. Other PPI meetings were funnelled to work specifically with people living with PD, HD and MS and subgroups met separately to voice disease‐related concerns. Meetings were conducted remotely and addressed different agendas: defining research questions, discussing the value of the project, refining data extraction templates, interpreting results and deciding on recommendations for successful integrated care interventions.

## 3 RESULTS

**Figure 1:**
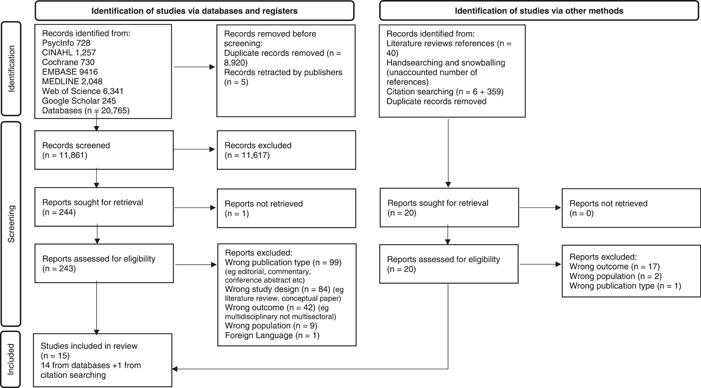
Preferred Reporting Items for Systematic Reviews and Meta‐Analyses flow‐chart for study selection.

Database search identified 20,765 articles (Figure 1), following deduplication this yielded 11,861 papers. A total of 11,617 articles were excluded on title and abstract screening. One reference could not be retrieved despite contacting the author institution and the journal editor. A total of 243 full‐text studies were screened and 229 excluded on full‐text screening leaving 14 eligible studies. One further study was identified through citation tracking of studies included in a literature review,^72^ resulting in a final total of 15 studies included in this review.^63^, ^64^, ^65^, ^73^, ^74^, ^75^, ^76^, ^77^, ^78^, ^79^, ^80^, ^81^, ^82^, ^83^, ^84^ Table 1 represents the study characteristics. The quality of studies was variable. Lower scores related to inexistent or very limited blinding of participants and assessors, lack of methodological clarity and gaps in rigour and data reporting. All studies had potential sources of bias (Supporting Information S1: Appendix 5).

**Table 1:** Characteristics of included studies.

Study/duration/country	Population condition and size (*n*)	Population characteristics	Study design	Sectors/levels of care^a^	Setting	Aim	Intervention(s)
Mestre et al. (2021)^63^ (6M) Canada	PD: 100 patients, 74 caregivers	Newly diagnosed. Advanced (not institutionalised).	Before–after	Primary care Secondary care	Community	Break silos in the healthcare system and increase accessibility to existing local care resources of interest to people with PD.	Integrated Parkinson Care Network (IPCN) Clinical Care Integrator (CCI)
Kessler et al. (2021)^64^ (6M) Canada	PD: 32 patients, 7 caregivers	Newly diagnosed. Advanced (not institutionalised).	Qualitative	Primary care Secondary care	Community	To evaluate the acceptability of the IPCN from the perspectives of persons with PD, their care partners and healthcare providers, including identification of important components and areas for improvement.	IPCN CCI
Connor et al. (2020)^65^ (18M/24M) USA	PD: 140 patients	Veterans (excluded dementia and unable to communicate).	RCT descriptive data	Private care Primary care Secondary care Social care	Specialist centre	To examine the quality (activities that occurred) and extent (frequency of these activities) during implementation of the Care Coordination for Health Promotion and Activities in Parkinson's Disease (CHAPS) protocol in a real‐world setting.	CHAPS Nurse care managers = care coordinator
Connor et al. (2019) (18M/24M) USA^73^	PD: 328 patients	Veterans (diagnosed >1 year).	RCT	Private care Primary care Secondary care Social care Third sector	Specialist centre	To test effects on care quality of chronic care model‐based Parkinson disease management.	CHAPS Nurse care managers = care coordinator
Munoz et al. (2020)^74^ (24M) Colombia	PD: 69 patients, 21 caregivers	Median years since diagnosis were 4 years, mainly male sample.	Postintervention survey	Private care Primary care Secondary care Public sector—University volunteers Third sector	Community	Document the experience of patients, caregivers, and experts in a community approach as an innovative model in a middle‐income country.	Saturdays‐in‐motion Clinical community approach
Fleisher et al. (2020)^75^ (12M) USA	PD: 27 patients	Advanced PD, homebound (excluded significant cognitive impairment).	Before–after	Secondary care Social care	Community	Determine whether facilitating expert in‐home care could improve our understanding of disease progression, treatment options and unmet needs in this vulnerable population, and whether such a model could mitigate decline in quality of life.	Interdisciplinary Home Visit Program for Advanced PD (HVP)
Fleisher et al. (2018)^76^ (29M) USA	PD: 67 patients HD: 1 patient	Advanced PD, homebound.	Retrospective	Primary care Secondary care Social care Third sector	Community	Detail a novel, interdisciplinary home visit program specifically designed for individuals with PD and related disorders and their family caregivers.	Interdisciplinary HVP Social worker = care coordinator
van der Marck et al. (2013) (8M)^77^ The Netherlands	PD: 301 patients, 196 caregivers	PD different stages (excluded cognitively impaired and comorbidity).	non‐RCT	Secondary care Tertiary care Social care	Specialist centre	Assess the effectiveness of an integrated multidisciplinary approach compared with usual care.	ParkinsonNet + expert centres
van der Marck et al. (2013)^84^ (8M) Canada	PD: 122 patients	PD different stages (excluded if dementia and no caregiver).	RCT	Secondary or tertiary care Social care	Specialist centre	Test if a multidisciplinary/specialist team offers better outcomes, compared to stand‐alone care from a general neurologist.	Expert multidisciplinary team (MDT) care and social care
Trend et al. (2022)^78^ (6W) UK	PD: 118 patients, 118 caregivers	PD different stages (excluded cognitively impaired and no caregiver) 50% H&Y 3.0.	Before–after	Secondary care Social care Third sector	Hospital	To evaluate the short‐term effectiveness of an intensive multidisciplinary rehabilitation programme for people with Parkinson's disease and their carers.	Intensive multidisciplinary rehabilitation programme Social worker = care coordinator
Healey et al. (2019)^79^ (12M) USA	MS: 21 patients	Severe disability, homebound, 70% female.	Before–after	Primary care Secondary care Third sector	Community	Report an initial evaluation of the Multiple Sclerosis at Home Access (MAHA) initiative, including measures of selected clinical quality outcomes, processes, results and collection of ongoing information.	MAHA MDT led comprehensivist = care coordinator
Jansen et al. (2006)^80^ (10M) The Netherlands	MS: 173 patients	40–50 years old, disease duration 9–12 years, majority female and married.	Quantitative	Primary care Secondary care Social care	Hospital	Assess a transmural care model for multiple sclerosis (TCMMS) patients to see whether it would improve patient outcomes, continuity of care and quality of life.	TCMMS MS nurse = care coordinator
Oeseburg et al. (2004)^81^ (15M) The Netherlands	MS: 40 patients	40–50 years old, disease duration 2–36 years.	Open case‐study	Primary care Secondary care Social care	Hospital	Present and evaluate a TCMMS.	TCMMS MS nurse = care coordinator
Zirra et al. (2017)^82^ (8W) UK	MS: 102 patients	74.5% Female 89% relapse remitting.	Prospective	Primary care Secondary care Tertiary care	Hospital	Assess the impact on health status of a multiple sclerosis (MS) nurse specialist telephone assessment/triage.	NeuroDirect integrated pathway MS nurse = care coordinator
Makepeace et al. (2001)^83^ (6M) UK	MS: 38 patients	Included cognitively impaired and no caregiver.	Prospective	Primary care Secondary care Social care Third sector	Community	Describes the introduction of a community multiple sclerosis team (CMST) in the city of Newcastle upon Tyne in the North of England.	CMST team member = care coordinator allocated to caseload
Total participants	HD: 1 MS: 374 PD: 1720 Total: 2095						

^a^Sectors/levels of care. Public sector: local government, the civil service, the NHS and higher education. Levels of health care: Primary care—GP, dentists and pharmacists. Secondary care—Community and hospital care. Planned care or urgent and emergency care. Tertiary care—Highly specialised treatment, for example, neurosurgery. Private sector: private and independent companies, organisations and consultancy firms. Third sector/voluntary care sector: Voluntary, community and social enterprise (charities).

Abbreviations: HD, Huntington's disease; M, months; MS, multiple sclerosis; *n*, number; PD, Parkinson's disease; RCT, randomized controlled trial; W, weeks

^a^ Sectors/levels of care.

### 3.1 Studies focus and nature of interventions

**Figure 2:**
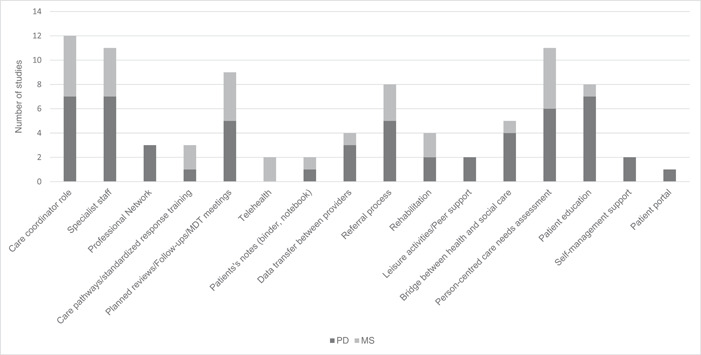
Characteristics of models of integrated care in the included literature.

Studies were delivered in five different countries: United States, Canada, The Netherlands, United Kingdom, and Colombia. Across the articles, a total of 11 interventions were identified for PD, 10 studies were identified, exploring seven interventions; for MS, five studies were identified, exploring four interventions; for HD, no studies were identified. Their length varied between 8 weeks and 29 months, with complex multilevel/multisectoral components, summarised in Figure 2. The most prevalent sector was public health represented by secondary care, often associated but not always with primary care. The least represented sector was private care, identified in only two interventions.^65^, ^73^, ^74^

Many studies reported having a care coordinator or specialist staff delivering care. Ten of the interventions also assessed person‐centred care needs, although the extent of the assessments varied between studies—interventions focused on patients' medical and psychosocial assessments,^73^ with less including house/safety/environment and financial needs.^76^ Some characteristics were harder to clarify due to limited reporting, like data access, transfer between providers and updating of data. For example, Connor et al.^73^ mentioned a patient portal and a notebook, but it is unclear how/if other teams had access to the system, how each tool was used and how the care plan was updated.

### 3.2 Studies outcomes

The review identified an extensive range of outcomes from the included literature summarised in Table 2 (Supporting Information S1: Appendix 6). Integrated care evidence was stronger for three outcomes: improved/decreased depression in patients, no impact in caregiver's burden and improved people's access to resources. UK studies indicated an improvement in patients' quality of life.

**Table 2:** Outcomes' strength of evidence in UK and non‐UK included studies.

Outcome	UK studies **Comparative design**	Strength of evidence rating	Non‐UK Comparative design	Strength of evidence rating	Non‐UK other studies design	Strength of evidence rating	Overall rating
Patients							
Levels of functioning/disability/independence	=^83^ −^83^ −^83^, a	Inconsistent evidence	+^84^ +^63^ +^75^ =^75^, b =^77^ =^73^	Inconsistent evidence	=^81^ =^81^ =^81^	Weaker evidence	Inconsistent evidence
Motor symptoms	+^78^, c	Very limited evidence	+^63^, c +^75^, c +^84^, c =^63^ =^75^ =^77^, c	Inconsistent evidence			Inconsistent evidence
Motor complications			=^75^	Very limited evidence			Very limited evidence
Nonmotor symptoms			=^63^ +^75^ +^73^	Inconsistent evidence			Inconsistent evidence
Nonmotor complications			+^73^ =^75^	Inconsistent evidence			Inconsistent evidence
Cognition			=^75^	Very limited evidence			Very limited evidence
Depression	+^78^	Very limited evidence	+^84^ +^73^ =Severe depression 73	Inconsistent evidence			Stronger evidence
Mental well‐being	=^83^	Very limited evidence	=^73^	Very limited evidence			Weaker evidence
Health related QoL			=^73^ +^80^	Inconsistent evidence	=^81^	Very limited evidence	Inconsistent evidence
QoL	+^78^ +^82^	Weaker evidence	=^75^ =^77^ +^84^	Inconsistent evidence			Inconsistent evidence
Education/information			=^73^ +^73^	Inconsistent evidence			Inconsistent evidence
Self‐management/self‐efficacy			+^63^ =^73^	Inconsistent evidence			Inconsistent evidence
Unmet needs identified			+^84^ +^73^, d =^73^ social	Inconsistent evidence			Inconsistent evidence
Needs met			+^80^ motor/personal care	Very limited evidence			Very limited evidence
Collaboration between providers			+^73^	Very limited evidence			Very limited evidence
Continuity of care			=^80^ +^73^	Inconsistent evidence			Inconsistent evidence
Perceived care			+^63^ =^73^	Inconsistent evidence			Inconsistent evidence
Caregivers							
Depression	=^78^	Very limited evidence					Very limited evidence
Burden			−^75^ =^63^ =^77^ =^84^	Stronger evidence			Stronger evidence
QoL	=^78^	Very limited evidence					Very limited evidence
Resource use and system impact							
Consultant appointments			−^80^	Very limited evidence			Very limited evidence
Specialist nurse appointments			+^80^	Very limited evidence			Very limited evidence
GP appointments			−^80^	Very limited evidence			Very limited evidence
Access to other resources			+^80^ +^73^	Stronger evidence			Stronger evidence
Healthcare utilization			=^75^	Very limited evidence			Very limited evidence
A&E visits			=^79^	Very limited evidence	+^76^	Very limited evidence	Very limited evidence
Hospitalizations			=^79^	Very limited evidence	+^76^ =^85^	Inconsistent evidence	Inconsistent evidence
Length of stay in hospital	+^83^	Very limited evidence	=^79^	Very limited evidence			Very limited evidence
Nursing home admissions			+^79^	Very limited evidence			Very limited evidence
Length of stay in nursing home			+^79^	Very limited evidence			Very limited evidence

*Note*: “+”, The study reported an improvement for this outcome; “=”, The study reported no significant change for this outcome; “−”, The study reported a decline/worsening for this outcome.

Abbreviations: GP, general practitioner; QoL, quality of life.

^a^ Newcastle independence scale showed no difference while Expanded Disability Status Scale and Functional independence measure showed decline.

^b^ Hoehn and Yahr not statistically significant, while Unified Parkinson Disease Rating Scale total score was statistically significant.

^c^ Physician examination.

^d^ Assessment of motor complications (i.e., on/off) for management, identification of motor complications.

#### 3.2.1 Patient outcomes

Most studies focused on reporting patient outcomes, particularly clinical outcomes, related to disease progression and motor symptoms,^63^, ^73^, ^75^, ^77^, ^78^, ^81^, ^83^, ^84^ with varied scales and outcomes measured. Their impact presented inconclusive evidence, with a mixture of improvement, no significance and decline. Nonmotor symptoms, mental well‐being, health related quality of life and quality of life were less reported, with inconsistent evidence. Nonmotor symptoms and health‐related quality of life were assessed in international literature but not in UK studies. Depression was assessed in only one UK study,^78^ showing improvement, which was supported by international evidence^73^, ^84^ showing strong evidence. Quality of life was assessed in two UK studies^78^, ^82^ showing an improvement. Internationally there was inconsistent evidence and overall, it remained inconsistent. Other outcomes: education, information received, self‐management/self‐efficacy; unmet needs identified and needs met; collaboration between providers; continuity of care; and perceived care were found in only four international studies^63^, ^73^, ^80^, ^84^ and neither in United Kingdom. These studies presented inconsistent or very limited evidence. Needs being met^80^ showed improvement for motor and personal care needs. Collaboration between providers^73^ showed improvement, and continuity of care and perceived care showed both improvement and no significance.^63^, ^73^, ^80^

#### 3.2.2 Caregiver outcomes

A few studies assessed caregivers' outcomes, reporting on depression, burden, and quality of life. Indeed 71% of the studies did not assess caregiver's outcomes. However, the few that did,^63^, ^75^, ^77^, ^78^, ^84^ revealed high and increasing burden. Depression and quality of life was only reported by Trend et al.^78^ and showed no difference. Burden was the most assessed outcome for caregivers, as reported in four international studies. Three studies^63^, ^77^, ^84^ showed no difference and one^75^ showed that caregiver's burden worsened through the study. In Trend et al.'s^78^ paper caregiver's burden was high with 10% of caregivers found in danger of being unable to continue caring. Through the programme carer's strain remained unchanged. In Fleisher et al.'s^75^ studycaregivers' strain increased mild to moderate after 1 year in the study, and some that withdrew were under severe strain. Munoz et al.^74^ assessed caregiver's burden postintervention but without baseline data it was not possible to establish its impact.

#### 3.2.3 Resource use and system outcomes

Outcomes related to resources use/system impact were varied: frequency of appointments, access to resources and healthcare utilization. Most of the outcomes assessed showed very limited evidence, being reported by individual studies which did not allow for a comparison across the literature. Only one UK study^83^ reported on this category, showing a reduction in length of stay in hospital; conversely one international study^79^ showed no difference. Admissions to the hospital were assessed in three studies^76^, ^79^, ^85^ but showed inconsistent evidence, with two studies showing no difference and one^76^ showing a reduction. The increase or decrease of appointments with different professionals could be seen as positive or negative depending on how care was perceived by people. However, most studies that reported on healthcare utilization did not report on patients' experience. Only one outcome showed strong evidence in this category: improved access to other resources, reported by two international studies.^73^, ^80^

### 3.3 Integrated care programmes characteristics

#### 3.3.1 Facilitating factors

When exploring the characteristics of the integrated care programmes^73^, ^78^, ^80^, ^82^, ^84^ that shown higher certainty (stronger and weaker strength) of improving people's outcomes, these had in common all four characteristics:

1. Specialist staff leading care. The teams were led by specialist staff in their disease‐related areas, hosted at hospitals and specialist centres.
2. Person‐centred care. The interventions focused on participants' specific needs and towards developing a personalised care plan.
3. Coordination of care. These interventions involved a care coordinator responsible for delivering the care plan and follow‐ups. The role was mostly performed by nurses, but some studies had a social care worker or used a dedicated specialist team to navigate care.
4. Continuity of care. All these interventions were characterised by planned reviews and follow‐ups.

#### 3.3.2 Hindering factors

When exploring the characteristics of the integrated care programmes that did not show significant differences or show decline in people's outcomes,^77^, ^81^, ^83^ it became evident that these studies focused on assessing clinical outcomes. For example, Oeseburg et al.^81^ reported meeting the needs of 2/3 of the participants and a reduction in people's needs. However, the primary outcomes selected did not reflect the positive impact of the programme. The same occurred on Makepeace et al.,^83^ where despite clinical outcomes pointing to disease progression, data related to living with the disease did not report worsening, suggesting better living with MS. Patients highlighted improved accessibility to resources and continuity of care. Although Makepeace et al.^83^ focused on motor/functional assessments, it did report on psychological wellbeing, which was crucial to understand its positive impact. Without this, its impact would have been missed. Furthermore, these programmes had feasibility issues with coordination and continuity of care. For example, van der Marck et al.^77^ lacked continuity of care by failing to schedule follow‐ups or to have a care coordinator to review/action the care plan as needed. Similarly, Oeseburg et al.^81^ faced obstacles on data transfer between providers.

## 4 DISCUSSION

In this systematic review, key characteristics of integrated care programmes that resulted in better outcomes for people living with PD, MS and HD included 1. Expert knowledge; 2. multisectoral care coordination; 3. care continuity and, 4. person‐centred approach. This review also identified several obstacles to integration including issues with data access and transitions between providers and found that integrated care impact on service users remains understudied.

### 4.1 Peoples' needs versus outcomes assessed

Our review shows that despite the complexity of interventions evaluated, the impact on people remains uncertain. The lack of research on the impact of integrated care on service users had been previously reported.^52^ Parker et al.^52^ found that patient outcomes, related to personal choice, empowerment, or continuity of care were largely absent from studies. This is consistent with this review results; despite people living with PD, MS and HD reporting common needs asking for better person‐centred integrated care, the most common outcomes assessed in the literature are clinical outcomes. We found strong evidence of reduction in patient's depression and improved access to resources, but other important outcomes matching people's care needs remained largely untested. Continuity of care was mostly untested despite its importance, but disease progression remained consistently tested despite the context of incurable neurodegeneration. Similarly, our review shows that caregivers' needs remain unmet despite being under severe distress.^75^, ^78^ When carers views were considered^63^, ^75^, ^77^, ^78^, ^84^ it was often unclear on how caregivers' needs were identified and addressed. This was not surprising considering that up to 85% of caregivers reported that their needs had not been assessed.^37^ Several studies^13^, ^17^, ^18^, ^24^, ^30^, ^32^ highlighted factors caregivers consider unhelpful, namely: lack of knowledge from staff, too many different case managers, no systematic screening of social care needs, and lack of financial assistance. According to these studies, better support would need to include increased access to respite care, better staff education and increased public awareness about the condition. Comparing these needs with the interventions tested in this review shows a clear mismatch. None of the interventions designed to date considered respite care access or any staff/public education. It is argued that delivering interventions that do not target or include caregivers' needs is ineffective, particularly in relation to carers' burden and quality of life outcomes.

### 4.2 Contributing evidence to people living with HD

HD is one of the most complex LTNCs^26^, ^37^ at individual and familial levels (people may struggle with keeping a social network of support, live with the stigma of psychiatric illness, lack cognitive capacity and caregivers experience high burden). Nevertheless, no literature reviews or empirical research were found about integrated care and HD; our review only identified one patient with HD^76^ amongst 2095 participants included. Through our search strategy we did find a relevant service evaluation by Veenhuizen et al.^86^ worth reflecting on due to showcasing the myriad of sectors involved in HD care (probation officers, municipal officials and regulation officers, etc.). The project,^86^ published 12 years ago, is still the most recent literature on integrated multisectoral care in HD. The intervention, a HD outreach clinic, promoted a proactive care approach, with biopsychosocial and environmental assessments, planned follow‐ups, personalised care plans, multisectoral collaboration and education of service users and service providers by the expert multidisciplinary team. These characteristics match the key characteristics of integrated care programmes identified in this review. Through a survey, patients reported quality of life improvement and caregivers reported good support from the expert team. Their findings were limited by a lack of comparative design and a lack of standardized evaluation tools, but they do suggest promising results. In the face of lack of evidence in the field of HD associated with complex health and care needs, it would be important that integrated care models are developed and tested in this underserved group. Aside from highlighting this gap, below our review provides considerations for future intervention development.

### 4.3 Recommendations in developing new interventions

The findings from this review can support the development of future integrated care interventions. We found that operational aspects like data centralization and transfer of data between professionals were overlooked and require attention in the development of future interventions at the risk of contributing to fragmentation. Lack of data sharing is a known barrier to integration and people cannot move between services and sectors seamlessly,^44^ a universally recognised problem across any country or condition. Hindering factors should be addressed to increase the success of future interventions.

In contrast, expert staff, good coordination between multisectoral providers, continuity of care and person‐centred approach are essential pillars that result in improved outcomes. These suggestions are consistent with previous literature,^44^, ^87^, ^88^, ^89^ highlighting that how teams operate in supporting people require a degree of maturity and operational comprehensiveness. These pillars should be taken into consideration by stakeholders and policymakers when designing, testing, and implementing new integrated care interventions.

### 4.4 Research impact

The findings suggest a discrepancy between people's needs and what programmes currently offer and the outcomes that are being assessed, questioning if current guidelines and integrated care policy are fit for purpose. Despite recommendations of integrated care to manage patients' complex needs, what success looks like still remains unclear. Studies that evaluated integrated care measured and reported varied outcomes (from Newcastle Independence Scale, to Expanded Disability Status Scale and Functional Independence Measure^83^ to Hoehn and Yahr and Unified Parkinson Disease Rating Scale^75^), making it difficult to compare results. This shows a need for further discussions around the core outcomes that matter most to people to explore if integrated care programmes actually benefit the intended end users. Methodological consensus regarding what aspects of integrated care should be measured would allow future researchers and clinicians to make sense of all the knowledge produced and thus improve the rate of progress in developing interventions. The WHO also recently acknowledged the need to develop a core set of indicators and targets to monitor national multisectoral action plans for intersectoral global action on neurological disorders.^90^ To advance integrated care for patient benefit, user‐driven outcomes that reflect person‐centred care are a potential solution^91^, ^92^; this will require involving patients and caregivers throughout the design stages to ensure relevance to users,^93^, ^94^, ^95^ instead of systems/organisations.^96^ While new measures of people's experiences of care are being developed,^97^ there is much more to be done to effectively understand the challenges that patients and caregivers face in negotiating the maze of services, organisations and funding and use this knowledge to deliver better care.^98^ Indeed, this strikes an important chord highlighting that successful integrated care interventions require multisectoral change (e.g., increase access to respite care) while focussing on person‐centred long‐term outcomes to capture their impact at user‐level (e.g., burden).

### 4.5 Strengths and limitations

The strengths of this review include an analysis of evidence strength from the perspective of patients' and caregivers' outcomes. Moreover, considers knowledge across three LTNCs, guided by patient and public contributors, adding value to the research conducted. However, one could argue that this focussed search on integrated care programmes for people living with PD, MS and HD as exemplar conditions, could be considered a limitation as it potentially excluded other noteworthy programmes or conditions. Considering there are hundreds of LTNCs it is acknowledged that this review represents a fraction on this field.

Our database search was conducted in 2021, meaning more recent papers may have been missed. To reflect on this limitation we used Cochrane's^99^ guiding checklist of when and how to update systematic reviews, considering that systematic reviews are time and resource consuming. We rerun our search strategy in MEDLINE and Google Scholar on the 9 October 2023 and did not find any papers published in this 2‐year period that would change our findings and conclusions. We did identify promising studies^100^, ^101^ currently being conducted in PD that, depending on their results, may prompt the need to update this systematic review in the future.

Our data extraction was primarily led by one author (S. B. P.), this may have introduced some level of researcher bias. Steps were taken to reduce this bias by independently testing the data extraction tool and several discussions took place amongst the researchers (S. B. P., M. C. P., D. K.) through the data extraction period. Lastly, the evidence grading presented some difficulties due to the heterogeneity of outcomes assessed and scales used; this was mitigated by reporting individually all outcomes which categorization was not straightforward and rating them individually before rating them across the literature, for transparency.

## 5 CONCLUSIONS

To date most multisectoral integrated care programmes have been primarily assessed through clinical outcomes. This medical‐centric perspective does not match people's most important care needs. People with PD and MS may benefit from better access to care and reduced depression but needs of caregivers and those living with more complex conditions like HD have been overlooked. There is the need to rethink how integrated care programmes are designed and evaluated to maximise the opportunity for positive change to update policies and improve people's outcomes. Multisectoral integrated care programmes for people and caregivers living with LTNCs should be investigated in a randomized controlled trial, once person‐centred outcomes that matter to them have been agreed upon or developed.

## AUTHOR CONTRIBUTIONS

**Sandra Bartolomeu Pires**: Methodology (lead); data curation; formal analysis; investigation; validation (equal); writing—original draft (lead); visualization (lead); writing—review and editing (lead). **Dorit Kunkel**: Conceptualization (supporting); methodology (supporting); formal analysis and investigation (equal); validation (equal); writing—original draft (equal); and writing—review and editing (equal). **Christopher Kipps**: Conceptualization (supporting); supervision (supporting); writing—review and editing (equal). **Nicholas Goodwin**: Conceptualization (supporting); supervision (supporting); writing—review and editing (equal). **Mari C. Portillo**: Conceptualization (lead); supervision and funding acquisition (lead); methodology (supporting); formal analysis and investigation (equal); validation (equal); writing—original draft (equal); and writing—review and editing (equal).

## CONFLICT OF INTEREST STATEMENT

The authors declare no conflicts of interest.

## Supporting information

Supporting information.

## Data Availability

The data that support the findings of this study are available from the corresponding author upon reasonable request.
