# Supplementary material for: Person‐centred integrated care for people living with Parkinson's, Huntington's and Multiple Sclerosis: A systematic review
Source: Health Expect. 2023 Dec 27;27(1):e13948. doi: 10.1111/hex.13948 (PMC10768870; doi:10.1111/hex.13948)
Supplement: Supplementary file 1 — Supporting information. [file HEX-27-e13948-s001.docx]

**Additional Files**

**Additional Files**

Person-centred integrated care for people living with Parkinson's, Huntington's and Multiple Sclerosis: A systematic review

**Contents:**

1. PRISMA checklist
2. Search strategies for databases
3. Search strategies for grey literature
4. Data extraction tool template
5. Risk of bias assessments
6. Outcomes table of included studies
7. References

# **Appendix 1. PRISMA checklist**

| **Section/topic** | **#** | **Checklist item** | **Reported on page #** |
| --- | --- | --- | --- |
| **TITLE** | | |  |
| Title | 1 | Identify the report as a systematic review, meta-analysis, or both. | 1 |
| **ABSTRACT** | | |  |
| Structured summary | 2 | Provide a structured summary including, as applicable: background; objectives; data sources; study eligibility criteria, participants, and interventions; study appraisal and synthesis methods; results; limitations; conclusions and implications of key findings; systematic review registration number. | 1 |
| **INTRODUCTION** | | |  |
| Rationale | 3 | Describe the rationale for the review in the context of what is already known. | 1 |
| Objectives | 4 | Provide an explicit statement of questions being addressed with reference to participants, interventions, comparisons, outcomes, and study design (PICOS). | 2 and PROSPERO protocol |
| **METHODS** | | |  |
| Protocol and registration | 5 | Indicate if a review protocol exists, if and where it can be accessed (e.g., Web address), and, if available, provide registration information including registration number. | 2 |
| Eligibility criteria | 6 | Specify study characteristics (e.g., PICOS, length of follow-up) and report characteristics (e.g., years considered, language, publication status) used as criteria for eligibility, giving rationale. | 2 |
| Information sources | 7 | Describe all information sources (e.g., databases with dates of coverage, contact with study authors to identify additional studies) in the search and date last searched. | 2 |
| Search | 8 | Present full electronic search strategy for at least one database, including any limits used, such that it could be repeated. | Appendix 2 |
| Study selection | 9 | State the process for selecting studies (i.e., screening, eligibility, included in systematic review, and, if applicable, included in the meta-analysis). | 4, Fig.1 |
| Data collection process | 10 | Describe method of data extraction from reports (e.g., piloted forms, independently, in duplicate) and any processes for obtaining and confirming data from investigators. | 3 |
| Data items | 11 | List and define all variables for which data were sought (e.g., PICOS, funding sources) and any assumptions and simplifications made. | 3,4 |
| Risk of bias in individual studies | 12 | Describe methods used for assessing risk of bias of individual studies (including specification of whether this was done at the study or outcome level), and how this information is to be used in any data synthesis. | Appendix 5 |
| Summary measures | 13 | State the principal summary measures (e.g., risk ratio, difference in means). | 3 |
| Synthesis of results | 14 | Describe the methods of handling data and combining results of studies, if done, including measures of consistency (e.g., I^2^) for each meta-analysis. | 3, Appendix 4 and 6 |
| **Section/topic** | **#** | **Checklist item** | **Reported on page #** |
| Risk of bias across studies | 15 | Specify any assessment of risk of bias that may affect the cumulative evidence (e.g., publication bias, selective reporting within studies). | Appendix 5 and p.6 |
| Additional analyses | 16 | Describe methods of additional analyses (e.g., sensitivity or subgroup analyses, meta-regression), if done, indicating which were pre-specified. | 3, 4, Appendix 4 and 6 |
| **RESULTS** | | |  |
| Study selection | 17 | Give numbers of studies screened, assessed for eligibility, and included in the review, with reasons for exclusions at each stage, ideally with a flow diagram. | 4 Fig.1 |
| Study characteristics | 18 | For each study, present characteristics for which data were extracted (e.g., study size, PICOS, follow-up period) and provide the citations. | 5, 6 Table 1 |
| Risk of bias within studies | 19 | Present data on risk of bias of each study and, if available, any outcome level assessment (see item 12). | Appendix 5 and p.6 |
| Results of individual studies | 20 | For all outcomes considered (benefits or harms), present, for each study: (a) simple summary data for each intervention group (b) effect estimates and confidence intervals, ideally with a forest plot. | Appendix 6 |
| Synthesis of results | 21 | Present the main results of the review. If meta-analyses are done, include for each, confidence intervals and measures of consistency | 5-12 |
| Risk of bias across studies | 22 | Present results of any assessment of risk of bias across studies (see Item 15). | Appendix 5 and p.6 |
| Additional analysis | 23 | Give results of additional analyses, if done (e.g., sensitivity or subgroup analyses, meta-regression [see Item 16]). | 11,12 |
| **DISCUSSION** | | |  |
| Summary of evidence | 24 | Summarize the main findings including the strength of evidence for each main outcome; consider their relevance to key groups (e.g., healthcare providers, users, and policy makers). | 12 |
| Limitations | 25 | Discuss limitations at study and outcome level (e.g., risk of bias), and at review-level (e.g., incomplete retrieval of identified research, reporting bias). | 13 |
| Conclusions | 26 | Provide a general interpretation of the results in the context of other evidence, and implications for future research. | 13 |
| **FUNDING** | | |  |
| Funding | 27 | Describe sources of funding for the systematic review and other support (e.g., supply of data); role of funders for the systematic review. | 13 (funding sources) |

*From:*  Moher D, Liberati A, Tetzlaff J, Altman DG, The PRISMA Group (2009). Preferred Reporting Items for Systematic Reviews and Meta-Analyses: The PRISMA Statement. PLoS Med 6(7): e1000097. doi:10.1371/journal.pmed1000097 ^2^

For more information, visit: **www.prisma-statement.org**.

Page 2 of 2

# **Appendix 2. Search strategies for the different databases ran from 02/09/2021-03/09/2021.**

We developed the search strategy with the assistance of two Librarians experienced with systematic reviews (VF and PS). Two members of the team provided feedback to the search strategy (MCP and DK). The strategies combined medical subject headings (MeSH) and keywords for the following concepts related to conditions under study and integrated care related terms.

**Table S-1.** Search strategy

| **Condition [Title and Abstract]** |  | **Integrated care [Title and Abstract]** | |
| --- | --- | --- | --- |
| **OR** | **AND** | **OR** | |
| Parkinson*  Huntington*  “Multiple sclerosis” |  | “Integrated care”  “Guided care”  “Manage* care”  “Shar* care”  “Holistic care”  “Seamless care”  “comprehensive care”  “Transmural care”  “Trans-mural care”  “coordinat* care”  “care coordination”  “co-ordinat* care”  “collaborat* care”  “continuum of care”  “care continuity”  “integrated healthcare”  “integrated health care”  “Care management”  “care pathway*”  “care-pathway*”  Integra*  Cooperati*  “joined-up”  “joined up”  “Co-operati*”  Multiagenc*  “Multi-agenc*” | Multisector*  “Multi-sector*”  Intersector*  “Inter-sector*”  Multidisciplinar*  “Multi-disciplinar*”  Interdisciplinar*  “Inter-disciplinar*”  Interprofessional  “Inter-professional”  “Multiprofessional”  “Multi-professional”  Transdisciplinar*  “Trans-disciplinar*”  Interorgani?ational  “Inter-organi?ational”  “Person cent*”  “Person-cent*”  “Patient cent*”  “Patient-cent*” |

The asterisk indicates the possible endings of some words.

Terms used for database search:

parkinson*.ti,ab.

huntington*.ti,ab.

multiple sclerosis.ti,ab.

Parkinson disease/

Huntington chorea/

multiple sclerosis/

“integra* health” N3 care.ti,ab.

integra*W3 prov*.ti,ab.

Integrat* W3 health W3 social.ti,ab.

"care manage*".ti,ab.

“case manag*”.ti,ab.

“care pathway*”.ti,ab.

“care-pathway*”.ti,ab.

care W3 model*.ti,ab.

model* W3 service*.ti,ab.

(model* W3 (“health-care” OR “healthcare”)).ti,ab.

(integrat* W3 (care OR “health-care” OR healthcare OR service* OR system* OR team* OR work* OR partner* OR collaboration* OR co-ordinat* OR coordinat*)).ti,ab.

(guided W3 (care OR “health-care” OR healthcare OR service* OR system* OR team* OR work* OR partner* OR collaboration* OR co-ordinat* OR coordinat*)).ti,ab.

(manage* W3 (care OR “health-care” OR healthcare OR service* OR system* OR team* OR work* OR partner* OR collaboration* OR co-ordinat* OR coordinat*)).ti,ab.

(shar* W3 (care OR “health-care” OR healthcare OR service* OR system* OR team* OR work* OR partner* OR collaboration* OR co-ordinat* OR coordinat*)).ti,ab.

(holistic W3 (care OR “health-care” OR healthcare OR service* OR system* OR team* OR work* OR partner* OR collaboration* OR co-ordinat* OR coordinat*)).ti,ab.

(seamless W3 (care OR “health-care” OR healthcare OR service* OR system* OR team* OR work* OR partner* OR collaboration* OR co-ordinat* OR coordinat*)).ti,ab.

(comprehensive W3 (care OR “health-care” OR healthcare OR service* OR system* OR team* OR work* OR partner* OR collaboration* OR co-ordinat* OR coordinat*)).ti,ab.

(continu* W3 (care OR “health-care” OR healthcare OR service* OR system* OR team* OR work* OR partner* OR collaboration* OR co-ordinat* OR coordinat*)).ti,ab.

(transmural W3 (care OR “health-care” OR healthcare OR service* OR system* OR team* OR work* OR partner* OR collaboration* OR co-ordinat* OR coordinat*)).ti,ab.

(“trans-mural” W3 (care OR “health-care” OR healthcare OR service* OR system* OR team* OR work* OR partner* OR collaboration* OR co-ordinat* OR coordinat*)).ti,ab.

(“trans mural” W3 (care OR “health-care” OR healthcare OR service* OR system* OR team* OR work* OR partner* OR collaboration* OR co-ordinat* OR coordinat*)).ti,ab.

(coordinat* W3 (care OR “health-care” OR healthcare OR service* OR system* OR team* OR work* OR partner* OR collaboration* OR co-ordinat* OR coordinat*)).ti,ab.

(“co-ordinat*” W3 (care OR “health-care” OR healthcare OR service* OR system* OR team* OR work* OR partner* OR collaboration* OR co-ordinat* OR coordinat*)).ti,ab.

(collaborat* W3 (care OR “health-care” OR healthcare OR service* OR system* OR team* OR work* OR partner* OR collaboration* OR co-ordinat* OR coordinat*)).ti,ab.

(cooperati* W3 (care OR “health-care” OR healthcare OR service* OR system* OR team* OR work* OR partner* OR collaboration* OR co-ordinat* OR coordinat*)).ti,ab.

(co-operati* W3 (care OR “health-care” OR healthcare OR service* OR system* OR team* OR work* OR partner* OR collaboration* OR co-ordinat* OR coordinat*)).ti,ab.

(“joined-up” W3 (care OR “health-care” OR healthcare OR service* OR system* OR team* OR work* OR partner* OR collaboration* OR co-ordinat* OR coordinat*)).ti,ab.

(“joined up” W3 (care OR “health-care” OR healthcare OR service* OR system* OR team* OR work* OR partner* OR collaboration* OR co-ordinat* OR coordinat*)).ti,ab.

(“join*” W3 (care OR “health-care” OR healthcare OR service* OR system* OR team* OR work* OR partner* OR collaboration* OR co-ordinat* OR coordinat*)).ti,ab.

(“personali?ed” W3 (care OR “health-care” OR healthcare OR service* OR system* OR team* OR work* OR partner* OR collaboration* OR co-ordinat* OR coordinat*)).ti,ab.

("person cent*" W3 (care OR “health-care” OR healthcare OR service* OR system* OR team* OR work* OR partner* OR collaboration* OR co-ordinat* OR coordinat*)).ti,ab.

(“person-cent*” W3 (care OR “health-care” OR healthcare OR service* OR system* OR team* OR work* OR partner* OR collaboration* OR co-ordinat* OR coordinat*)).ti,ab.

("patient cent*" W3 (care OR “health-care” OR healthcare OR service* OR system* OR team* OR work* OR partner* OR collaboration* OR co-ordinat* OR coordinat*)).ti,ab.

(“patient-cent* W3 (care OR “health-care” OR healthcare OR service* OR system* OR team* OR work* OR partner* OR collaboration* OR co-ordinat* OR coordinat*)).ti,ab.

(multiagenc* W3 (care OR “health-care” OR healthcare OR service* OR system* OR team* OR work* OR partner* OR collaboration* OR co-ordinat* OR coordinat*)).ti,ab.

(“multi-agenc*” W3 (care OR “health-care” OR healthcare OR service* OR system* OR team* OR work* OR partner* OR collaboration* OR co-ordinat* OR coordinat*)).ti,ab.

(“multi agenc*” W3 (care OR “health-care” OR healthcare OR service* OR system* OR team* OR work* OR partner* OR collaboration* OR co-ordinat* OR coordinat*)).ti,ab.

(interagenc* W3 (care OR “health-care” OR healthcare OR service* OR system* OR team* OR work* OR partner* OR collaboration* OR co-ordinat* OR coordinat*)).ti,ab.

(“inter-agenc*” W3 (care OR “health-care” OR healthcare OR service* OR system* OR team* OR work* OR partner* OR collaboration* OR co-ordinat* OR coordinat*)).ti,ab.

(“inter agenc*” W3 (care OR “health-care” OR healthcare OR service* OR system* OR team* OR work* OR partner* OR collaboration* OR co-ordinat* OR coordinat*)).ti,ab.

(multisect* W3 (care OR “health-care” OR healthcare OR service* OR system* OR team* OR work* OR partner* OR collaboration* OR co-ordinat* OR coordinat*)).ti,ab.

(“multi-sect*” W3 (care OR “health-care” OR healthcare OR service* OR system* OR team* OR work* OR partner* OR collaboration* OR co-ordinat* OR coordinat*)).ti,ab.

(“multi sect*” W3 (care OR “health-care” OR healthcare OR service* OR system* OR team* OR work* OR partner* OR collaboration* OR co-ordinat* OR coordinat*)).ti,ab.

(intersect* W3 (care OR “health-care” OR healthcare OR service* OR system* OR team* OR work* OR partner* OR collaboration* OR co-ordinat* OR coordinat*)).ti,ab.

(“inter-sect*” W3 (care OR “health-care” OR healthcare OR service* OR system* OR team* OR work* OR partner* OR collaboration* OR co-ordinat* OR coordinat*)).ti,ab.

(“inter sect*” W3 (care OR “health-care” OR healthcare OR service* OR system* OR team* OR work* OR partner* OR collaboration* OR co-ordinat* OR coordinat*)).ti,ab.

(multidisciplinar* W3 (care OR “health-care” OR healthcare OR service* OR system* OR team* OR work* OR partner* OR collaboration* OR co-ordinat* OR coordinat*)).ti,ab.

(“multi-disciplinar*” W3 (care OR “health-care” OR healthcare OR service* OR system* OR team* OR work* OR partner* OR collaboration* OR co-ordinat* OR coordinat*)).ti,ab.

(“multi disciplinar*” W3 (care OR “health-care” OR healthcare OR service* OR system* OR team* OR work* OR partner* OR collaboration* OR co-ordinat* OR coordinat*)).ti,ab.

(interdisciplinar* W3 (care OR “health-care” OR healthcare OR service* OR system* OR team* OR work* OR partner* OR collaboration* OR co-ordinat* OR coordinat*)).ti,ab.

(“inter-disciplinar*” W3 (care OR “health-care” OR healthcare OR service* OR system* OR team* OR work* OR partner* OR collaboration* OR co-ordinat* OR coordinat*)).ti,ab.

(“inter disciplinar*” W3 (care OR “health-care” OR healthcare OR service* OR system* OR team* OR work* OR partner* OR collaboration* OR co-ordinat* OR coordinat*)).ti,ab.

(transdisciplinar* W3 (care OR “health-care” OR healthcare OR service* OR system* OR team* OR work* OR partner* OR collaboration* OR co-ordinat* OR coordinat*)).ti,ab.

(“trans-disciplinar*” W3 (care OR “health-care” OR healthcare OR service* OR system* OR team* OR work* OR partner* OR collaboration* OR co-ordinat* OR coordinat*)).ti,ab.

(“trans disciplinar*” W3 (care OR “health-care” OR healthcare OR service* OR system* OR team* OR work* OR partner* OR collaboration* OR co-ordinat* OR coordinat*)).ti,ab.

(interprofession* W3 (care OR “health-care” OR healthcare OR service* OR system* OR team* OR work* OR partner* OR collaboration* OR co-ordinat* OR coordinat*)).ti,ab.

(“inter-profession*” W3 (care OR “health-care” OR healthcare OR service* OR system* OR team* OR work* OR partner* OR collaboration* OR co-ordinat* OR coordinat*)).ti,ab.

(“inter profession*” W3 (care OR “health-care” OR healthcare OR service* OR system* OR team* OR work* OR partner* OR collaboration* OR co-ordinat* OR coordinat*)).ti,ab.

(“multiprofession*” W3 (care OR “health-care” OR healthcare OR service* OR system* OR team* OR work* OR partner* OR collaboration* OR co-ordinat* OR coordinat*)).ti,ab.

(“multi-profession*” W3 (care OR “health-care” OR healthcare OR service* OR system* OR team* OR work* OR partner* OR collaboration* OR co-ordinat* OR coordinat*)).ti,ab.

(“multi profession*” W3 (care OR “health-care” OR healthcare OR service* OR system* OR team* OR work* OR partner* OR collaboration* OR co-ordinat* OR coordinat*)).ti,ab.

(“multispeciality” W3 (care OR “health-care” OR healthcare OR service* OR system* OR team* OR work* OR partner* OR collaboration* OR co-ordinat* OR coordinat*)).ti,ab.

(“multi-speciality” W3 (care OR “health-care” OR healthcare OR service* OR system* OR team* OR work* OR partner* OR collaboration* OR co-ordinat* OR coordinat*)).ti,ab.

(“multi speciality” W3 (care OR “health-care” OR healthcare OR service* OR system* OR team* OR work* OR partner* OR collaboration* OR co-ordinat* OR coordinat*)).ti,ab.

(Interorgani?ational W3 (care OR “health-care” OR healthcare OR service* OR system* OR team* OR work* OR partner* OR collaboration* OR co-ordinat* OR coordinat*)).ti,ab.

(“Inter-organi?ational” W3 (care OR “health-care” OR healthcare OR service* OR system* OR team* OR work* OR partner* OR collaboration* OR co-ordinat* OR coordinat*)).ti,ab.

(“Inter organi?ational” W3 (care OR “health-care” OR healthcare OR service* OR system* OR team* OR work* OR partner* OR collaboration* OR co-ordinat* OR coordinat*)).ti,ab.

Integrated care/

# **CINAHL 30/09/2021**

1. TI ( parkinson* OR huntington* OR "multiple sclerosis" ) OR AB ( parkinson* OR huntington* OR "multiple sclerosis" )

2. (MH "Parkinson Disease")

3. (MH "Huntington's Disease")

4. (MH "Multiple Sclerosis")

5. S1 OR S2 OR S3 OR S4

6. TI ( (“integra* health” N3 care) OR ((integra* N3 (prov* OR health OR social) OR ("care manage*") OR (“case manag*”) OR (“care-pathway*”) OR ( (model* N3 (“health-care” OR “healthcare” OR care OR service*)) )

7. AB ( (“integra* health” N3 care) OR ((integra* N3 (prov* OR health OR social) OR ("care manage*") OR (“case manag*”) OR (“care-pathway*”) OR ( (model* N3 (“health-care” OR “healthcare” OR care OR service*)) )

8. TI ( ((integrat* OR guided OR manage* OR shar* OR holistic OR seamless OR comprehensive OR continu* OR transmural OR “trans-mural” OR coordinat* OR “co-ordinat*” OR collaborat* OR cooperative* OR “co-operativ*” OR “joined-up” OR “join*” OR “personali?ed” OR “person-cent*” OR “patient-cent*” OR multiagenc* OR “multi-agenc*” OR interagenc* OR “inter-agenc*” OR multisect* OR “multi-sect*” OR intersect* OR “inter-sect*” OR multidisciplinar* OR “multi-disciplinar*” OR interdisciplinar* OR “inter-disciplinar*” OR transdisciplinar* OR “trans-disciplinar*” OR interprofession* OR “inter-profession*” OR “multiprofession*” OR “multi-profession*” OR “multispeciality” OR “multi-speciality” OR Interorgani?ational OR “Inter-organi?ational”) W3 (care OR healthcare OR “health-care” OR service* OR system* OR team* OR work* OR partner* OR collaboration* OR co-ordinat* OR coordinat*)) )

9. AB ( ((integrat* OR guided OR manage* OR shar* OR holistic OR seamless OR comprehensive OR continu* OR transmural OR “trans-mural” OR coordinat* OR “co-ordinat*” OR collaborat* OR cooperative* OR “co-operativ*” OR “joined-up” OR “join*” OR “personali?ed” OR “person-cent*” OR “patient-cent*” OR multiagenc* OR “multi-agenc*” OR interagenc* OR “inter-agenc*” OR multisect* OR “multi-sect*” OR intersect* OR “inter-sect*” OR multidisciplinar* OR “multi-disciplinar*” OR interdisciplinar* OR “inter-disciplinar*” OR transdisciplinar* OR “trans-disciplinar*” OR interprofession* OR “inter-profession*” OR “multiprofession*” OR “multi-profession*” OR “multispeciality” OR “multi-speciality” OR Interorgani?ational OR “Inter-organi?ational”) W3 (care OR healthcare OR “health-care” OR service* OR system* OR team* OR work* OR partner* OR collaboration* OR co-ordinat* OR coordinat*)) )

10. (MH "Health Care Delivery, Integrated") OR (MH "Clinical Information Systems")

11. 6 OR 7 OR 8 OR 9 OR 10

12. 5 AND 11 = 1345 FROM 2000 = 1,271 with language limits = 1,257

# **Cochrane 30/09/2021**

1. TI ( parkinson* OR huntington* OR "multiple sclerosis" ) OR AB ( parkinson* OR huntington* OR "multiple sclerosis" )

2. DE "Parkinson's Disease"

3. DE "Huntingtons Disease"

4. DE "Multiple Sclerosis"

5. S1 OR S2 OR S3 OR S4

6. ( (“integra* health” near/3 care): ti,ab,kw

7. (prov* OR health OR social) near/3 care): ti,ab,kw

8. (manag* OR pathway*) near/3 care): ti,ab,kw

9. ("care manage*"): ti,ab,kw

10. (“health-care” OR “healthcare” OR care OR service*) near/3 model): ti,ab,kw

11. ( ((integrat* OR guided OR manage* OR shar* OR holistic OR seamless OR comprehensive OR continu* OR transmural OR “trans-mural” OR coordinat* OR “co-ordinat*” OR collaborat* OR cooperative* OR “co-operativ*” OR “joined-up” OR “join*” OR “personali?ed” OR “person-cent*” OR “patient-cent*” OR multiagenc* OR “multi-agenc*” OR interagenc* OR “inter-agenc*” OR multisect* OR “multi-sect*” OR intersect* OR “inter-sect*” OR multidisciplinar* OR “multi-disciplinar*” OR interdisciplinar* OR “inter-disciplinar*” OR transdisciplinar* OR “trans-disciplinar*” OR interprofession* OR “inter-profession*” OR “multiprofession*” OR “multi-profession*” OR “multispeciality” OR “multi-speciality” OR Interorgani?ational OR “Inter-organi?ational”) near/3 (care OR healthcare OR “health-care” OR service* OR system* OR team* OR work* OR partner* OR collaboration* OR co-ordinat* OR coordinat*)) ) : ti,ab,kw

12. MH “integrated care”: [delivery of health care, integrated] explode all trees

13. 6 OR 7 OR 8 OR 9 OR 10 OR 11 OR 12

14. 5 AND 13 = 746 FROM 2000 = **730** with language limits = unable to apply

**Embase** 30/09/2021 1974 to 2021 Week 38

parkinson*.ti,ab.

huntington*.ti,ab.

multiple sclerosis.ti,ab.

Parkinson disease/

Huntington chorea/

multiple sclerosis/

1 or 2 or 3 or 4 or 5 or 6

((integra* health adj3 care) or ((prov* or health or social) adj3 integra*) or care manage* or case manag* or care-pathway* or ((health-care or healthcare or care or service*) adj3 model*)).ti,ab.

((integrat* or guided or manage* or shar* or holistic or seamless or comprehensive or continu* or transmural or trans-mural or coordinat* or co-ordinat* or collaborat* or cooperative* or co-operativ* or joined-up or join* or personali?ed or person-cent* or patient-cent* or multiagenc* or multi-agenc* or interagenc* or inter-agenc* or multisect* or multi-sect* or intersect* or inter-sect* or multidisciplinar* or multi-disciplinar* or interdisciplinar* or inter-disciplinar* or transdisciplinar* or trans-disciplinar* or interprofession* or inter-profession* or multiprofession* or multi-profession* or multispeciality or multi-speciality or Interorgani?ational or Inter-organi?ational) adj3 (care or healthcare or health-care or service* or system* or team* or work* or partner* or collaboration* or co-ordinat* or coordinat*)).ti,ab.

integrated health care system/

8 or 9 or 10

7 and 11

12 and 2000:2021.(sa_year).

limit 13 to english

limit 13 to german

limit 13 to portuguese

limit 13 to spanish

14 or 15 or 16 or 17

parkinson*.ti,ab.
huntington*.ti,ab.
multiple sclerosis.ti,ab.
Parkinson disease/
Huntington chorea/
multiple sclerosis/
1 or 2 or 3 or 4 or 5 or 6

((integra* health adj3 care) or ((prov* or health or social) adj3 integra*) or care manage* or case manag* or care-pathway* or ((health-care or healthcare or care or service*) adj3 model*)).ti,ab.

((integrat* or guided or manage* or shar* or holistic or seamless or comprehensive or continu* or transmural or trans-mural or coordinat* or co-ordinat* or collaborat* or cooperative* or co-operativ* or joined-up or join* or personali?ed or person-cent* or patient-cent* or multiagenc* or multi-agenc* or interagenc* or inter-agenc* or multisect* or multi-sect* or intersect* or inter-sect* or multidisciplinar* or multi-disciplinar* or interdisciplinar* or inter-disciplinar* or transdisciplinar* or trans-disciplinar* or interprofession* or inter-profession* or multiprofession* or multi-profession* or multispeciality or multi-speciality or Interorgani?ational or Inter-organi?ational) adj3 (care or healthcare or health-care or service* or system* or team* or work* or partner* or collaboration* or co-ordinat* or coordinat*)).ti,ab.

integrated health care system/
8 or 9 or 10
7 and 11
limit 12 to english
limit 12 to german
limit 12 to portuguese
limit 12 to spanish
13 or 14 or 15 or 16

7 AND 11 = 10162 FROM 2000 - 2021= 9602 with language limits = 9416

| **#** | **Query** | **Results from 29 Sep 2021** |
| --- | --- | --- |
| 1 | parkinson*.ti,ab. | 178,811 |
| 2 | huntington*.ti,ab. | 25,153 |
| 3 | multiple sclerosis.ti,ab. | 124,217 |
| 4 | Parkinson disease/ | 166,037 |
| 5 | Huntington chorea/ | 28,683 |
| 6 | multiple sclerosis/ | 139,071 |
| 7 | 1 or 2 or 3 or 4 or 5 or 6 | 378,629 |
| 8 | ((integra* health adj3 care) or ((prov* or health or social) adj3 integra*) or care manage* or case manag* or care-pathway* or ((health-care or healthcare or care or service*) adj3 model*)).ti,ab. | 125,193 |
| 9 | ((integrat* or guided or manage* or shar* or holistic or seamless or comprehensive or continu* or transmural or trans-mural or coordinat* or co-ordinat* or collaborat* or cooperative* or co-operativ* or joined-up or join* or personali?ed or person-cent* or patient-cent* or multiagenc* or multi-agenc* or interagenc* or inter-agenc* or multisect* or multi-sect* or intersect* or inter-sect* or multidisciplinar* or multi-disciplinar* or interdisciplinar* or inter-disciplinar* or transdisciplinar* or trans-disciplinar* or interprofession* or inter-profession* or multiprofession* or multi-profession* or multispeciality or multi-speciality or Interorgani?ational or Inter-organi?ational) adj3 (care or healthcare or health-care or service* or system* or team* or work* or partner* or collaboration* or co-ordinat* or coordinat*)).ti,ab. | 862,730 |
| 10 | integrated health care system/ | 12,195 |
| 11 | 8 or 9 or 10 | 933,972 |
| 12 | 7 and 11 | 10,162 |
| 13 | 12 and 2000:2021.(sa_year). | 9,602 |
| 14 | limit 13 to english | 9,278 |
| 15 | limit 13 to german | 94 |
| 16 | limit 13 to portuguese | 15 |
| 17 | limit 13 to spanish | 61 |
| 18 | 14 or 15 or 16 or 17 | 9,416 |

# **PSYCINFO 30/09/2021**

1. TI ( parkinson* OR huntington* OR "multiple sclerosis" ) OR AB ( parkinson* OR huntington* OR "multiple sclerosis" )

2. DE "Parkinson's Disease"

3. DE "Huntingtons Disease"

4. DE "Multiple Sclerosis"

5. S1 OR S2 OR S3 OR S4

6. TI ( (“integra* health” N3 care) OR ((integra* N3 (prov* OR health OR social) OR ("care manage*") OR (“case manag*”) OR (“care-pathway*”) OR ( (model* N3 (“health-care” OR “healthcare” OR care OR service*)) )

7. AB ( (“integra* health” N3 care) OR ((integra* N3 (prov* OR health OR social) OR ("care manage*") OR (“case manag*”) OR (“care-pathway*”) OR ( (model* N3 (“health-care” OR “healthcare” OR care OR service*)) )

8. TI ( ((integrat* OR guided OR manage* OR shar* OR holistic OR seamless OR comprehensive OR continu* OR transmural OR “trans-mural” OR coordinat* OR “co-ordinat*” OR collaborat* OR cooperative* OR “co-operativ*” OR “joined-up” OR “join*” OR “personali?ed” OR “person-cent*” OR “patient-cent*” OR multiagenc* OR “multi-agenc*” OR interagenc* OR “inter-agenc*” OR multisect* OR “multi-sect*” OR intersect* OR “inter-sect*” OR multidisciplinar* OR “multi-disciplinar*” OR interdisciplinar* OR “inter-disciplinar*” OR transdisciplinar* OR “trans-disciplinar*” OR interprofession* OR “inter-profession*” OR “multiprofession*” OR “multi-profession*” OR “multispeciality” OR “multi-speciality” OR Interorgani?ational OR “Inter-organi?ational”) W3 (care OR healthcare OR “health-care” OR service* OR system* OR team* OR work* OR partner* OR collaboration* OR co-ordinat* OR coordinat*)) )

9. AB ( ((integrat* OR guided OR manage* OR shar* OR holistic OR seamless OR comprehensive OR continu* OR transmural OR “trans-mural” OR coordinat* OR “co-ordinat*” OR collaborat* OR cooperative* OR “co-operativ*” OR “joined-up” OR “join*” OR “personali?ed” OR “person-cent*” OR “patient-cent*” OR multiagenc* OR “multi-agenc*” OR interagenc* OR “inter-agenc*” OR multisect* OR “multi-sect*” OR intersect* OR “inter-sect*” OR multidisciplinar* OR “multi-disciplinar*” OR interdisciplinar* OR “inter-disciplinar*” OR transdisciplinar* OR “trans-disciplinar*” OR interprofession* OR “inter-profession*” OR “multiprofession*” OR “multi-profession*” OR “multispeciality” OR “multi-speciality” OR Interorgani?ational OR “Inter-organi?ational”) W3 (care OR healthcare OR “health-care” OR service* OR system* OR team* OR work* OR partner* OR collaboration* OR co-ordinat* OR coordinat*)) )

10. DE "Integrated Services" OR DE "Continuum of Care" OR DE "Health Care Utilization" OR DE "Patient Centered Care"

11. 6 OR 7 OR 8 OR 9 OR 10

12. 5 AND 11 = 796 FROM 2000 = 740 with language limits = 728 (no papers in Portuguese or Spanish)

# **Medline on EBSCO 30/09/2021**

1. TI ( parkinson* OR huntington* OR "multiple sclerosis" ) OR AB ( parkinson* OR huntington* OR "multiple sclerosis" )

2. (MH "Parkinson Disease")

3. (MH "Huntington's Disease")

4. (MH "Multiple Sclerosis")

5. S1 OR S2 OR S3 OR S4

6. TI ( (“integra* health” N3 care) OR ((integra* N3 (prov* OR health OR social) OR ("care manage*") OR (“case manag*”) OR (“care-pathway*”) OR ( (model* N3 (“health-care” OR “healthcare” OR care OR service*)) )

7. AB ( (“integra* health” N3 care) OR ((integra* N3 (prov* OR health OR social) OR ("care manage*") OR (“case manag*”) OR (“care-pathway*”) OR ( (model* N3 (“health-care” OR “healthcare” OR care OR service*)) )

8. TI ( ((integrat* OR guided OR manage* OR shar* OR holistic OR seamless OR comprehensive OR continu* OR transmural OR “trans-mural” OR coordinat* OR “co-ordinat*” OR collaborat* OR cooperative* OR “co-operativ*” OR “joined-up” OR “join*” OR “personali?ed” OR “person-cent*” OR “patient-cent*” OR multiagenc* OR “multi-agenc*” OR interagenc* OR “inter-agenc*” OR multisect* OR “multi-sect*” OR intersect* OR “inter-sect*” OR multidisciplinar* OR “multi-disciplinar*” OR interdisciplinar* OR “inter-disciplinar*” OR transdisciplinar* OR “trans-disciplinar*” OR interprofession* OR “inter-profession*” OR “multiprofession*” OR “multi-profession*” OR “multispeciality” OR “multi-speciality” OR Interorgani?ational OR “Inter-organi?ational”) W3 (care OR healthcare OR “health-care” OR service* OR system* OR team* OR work* OR partner* OR collaboration* OR co-ordinat* OR coordinat*)) )

9. AB ( ((integrat* OR guided OR manage* OR shar* OR holistic OR seamless OR comprehensive OR continu* OR transmural OR “trans-mural” OR coordinat* OR “co-ordinat*” OR collaborat* OR cooperative* OR “co-operativ*” OR “joined-up” OR “join*” OR “personali?ed” OR “person-cent*” OR “patient-cent*” OR multiagenc* OR “multi-agenc*” OR interagenc* OR “inter-agenc*” OR multisect* OR “multi-sect*” OR intersect* OR “inter-sect*” OR multidisciplinar* OR “multi-disciplinar*” OR interdisciplinar* OR “inter-disciplinar*” OR transdisciplinar* OR “trans-disciplinar*” OR interprofession* OR “inter-profession*” OR “multiprofession*” OR “multi-profession*” OR “multispeciality” OR “multi-speciality” OR Interorgani?ational OR “Inter-organi?ational”) W3 (care OR healthcare OR “health-care” OR service* OR system* OR team* OR work* OR partner* OR collaboration* OR co-ordinat* OR coordinat*)) )

10. (MH "Delivery of Health Care, Integrated") OR (MH "Transitional Care")

11. 6 OR 7 OR 8 OR 9 OR 10

12. 5 AND 11 = 2,295 FROM 2000 = 2,117 with language limits = 2,048

**Web of Science 30/09/2021**

#1 (TI=(parkinson* OR huntington* OR "multiple sclerosis")) OR AB=(parkinson* OR huntington* OR "multiple sclerosis")

#2 (((TI=((((“integra* health”) NEAR/3 care))) )) OR AB=((((“integra* health”) NEAR/3 care))) )

#3 (((TI=((((prov* OR health OR social) NEAR/3 care))) )) OR AB=((((prov* OR health OR social) NEAR/3 care))) )

#4 (((TI=(((((manag* OR pathway*) NEAR/3 care)))) )) OR AB=(((((manag* OR pathway*) NEAR/3 care)))) )

#5 (((TI=((("case manage*"))) )) OR AB=((("case manage*"))) )

#6 (((TI=((((((“health-care” OR “healthcare” OR care OR service*) NEAR/3 model)))))) OR AB=((((((“health-care” OR “healthcare” OR care OR service*) NEAR/3 model)))))))

#7 (((TI=((( ((integrat* OR guided OR manage* OR shar* OR holistic OR seamless OR comprehensive OR continu* OR transmural OR “trans-mural” OR coordinat* OR “co-ordinat*” OR collaborat* OR cooperative* OR “co-operativ*” OR “joined-up” OR “join*” OR “personali$ed” OR “person-cent*” OR “patient-cent*” OR multiagenc* OR “multi-agenc*” OR interagenc* OR “inter-agenc*” OR multisect* OR “multi-sect*” OR intersect* OR “inter-sect*” OR multidisciplinar* OR “multi-disciplinar*” OR interdisciplinar* OR “inter-disciplinar*” OR transdisciplinar* OR “trans-disciplinar*” OR interprofession* OR “inter-profession*” OR “multiprofession*” OR “multi-profession*” OR “multispeciality” OR “multi-speciality” OR Interorgani$ational OR “Inter-organi$ational”) NEAR/3 (care OR healthcare OR “health-care” OR service* OR system* OR team* OR work* OR partner* OR collaboration* OR co-ordinat* OR coordinat*)) ))) )) OR AB=((( ((integrat* OR guided OR manage* OR shar* OR holistic OR seamless OR comprehensive OR continu* OR transmural OR “trans-mural” OR coordinat* OR “co-ordinat*” OR collaborat* OR cooperative* OR “co-operativ*” OR “joined-up” OR “join*” OR “personali$ed” OR “person-cent*” OR “patient-cent*” OR multiagenc* OR “multi-agenc*” OR interagenc* OR “inter-agenc*” OR multisect* OR “multi-sect*” OR intersect* OR “inter-sect*” OR multidisciplinar* OR “multi-disciplinar*” OR interdisciplinar* OR “inter-disciplinar*” OR transdisciplinar* OR “trans-disciplinar*” OR interprofession* OR “inter-profession*” OR “multiprofession*” OR “multi-profession*” OR “multispeciality” OR “multi-speciality” OR Interorgani$ational OR “Inter-organi$ational”) NEAR/3 (care OR healthcare OR “health-care” OR service* OR system* OR team* OR work* OR partner* OR collaboration* OR co-ordinat* OR coordinat*)) ))) )

#8 #2 or #3 or #4 or #5 or #6 or #7

#9 #1 AND #8

#10 YEAR 2000-2021

#11 #1 AND #8 and English or German or Spanish or Portuguese (Languages)

Results 6,807 from 2000 = 6,434 w/ language limits: Spanish, portuguese, german and English = **6,341**

**Google Scholar 30/09/2021**

With all of the words

allintitle: "integrated care" | "seamless care" | "comprehensive care" | "transmural care" | "coordinated care" parkinson's | huntington's | "multiple sclerosis" Results 48 articles (citations excluded, limits 2000-2021 language limits English, german, spanish and Portuguese.

allintitle: "continuum of care" | "collaborative care" | "care continuity" | "care pathway" | "care management" parkinson's | huntington's | "multiple sclerosis" Results 53

allintitle: "integrated healthcare" | "integrated health care" | “guided care” | “joined-up care” | “joined up care” | “multi-sectoral care” | “multisectoral care” | “inter-sectoral care” | “intersectoral care” parkinson's | huntington's | "multiple sclerosis" Results 4

allintitle: “multiagency care” | “multi-agency care” | “multidisciplinary care” | “multi-disciplinary care” | “interdisciplinary care” | “inter-disciplinary care” parkinson's | huntington's | "multiple sclerosis" Results 34

allintitle: interagency | “inter-agency” | “shared care” | “holistic care” | “cooperative care” | “co-operative care” parkinson's | huntington's | "multiple sclerosis" Results 1

allintitle: “interprofessional care” | “inter-professional care” | “multiprofessional care” | “multi-professional care” | “transdisciplinary care” | “trans-disciplinar care” parkinson's | huntington's | "multiple sclerosis" Results 2

allintitle: "Inter organisational" | "Inter organizational" | Inter-organisational | Inter-organizational parkinson's | huntington's | "multiple sclerosis" Results 0

allintitle: "person centred" | “person-centred” | "patient centred" | “patient-centred” parkinson's | huntington's | "multiple sclerosis" Results 14

allintitle: "person centered" | “person-centered” | "patient centered" | “patient-centered” parkinson's | huntington's | "multiple sclerosis" Results 49

allintitle: “care model” | “model of care” | “personalised care” | “personalized care” parkinson's | huntington's | "multiple sclerosis" Results 40

allintitle: “case manager” parkinson's | huntington's | "multiple sclerosis" Results 0

allintitle: “multi-profession*” | “multispeciality” | “multi-speciality” parkinson's | huntington's | "multiple sclerosis" Results 0

Total google scholar: 245

# **Appendix 3. Search strategies for grey literature ran from 08/05/2022 to 13/05/2022**

**Hand and citation searching and snowballing**

The journals below had alerts set up manually since 22/AUG/2020, some were covered by the search set up on Zetoc ^1^. Notifications were received regularly, and eligibility criteria was applied.

Zetoc alerts set on the 22/AUG/2020 with keywords: 1- "integrated care" parkinson huntington neurological intersectoral multisectoral 2 – “integrated care” “long term condition”; 3 – “integrated care” neuro; 4 – “"coordinated care" neuro; 5 - "integrated care" Parkinson; 6 - "integrated care" huntington

*Zetoc Alert - Display List Contents (22AUG2020)*

*List "Integrated care LTNCs" now contains these journals:*

*BMC HEALTH SERVICES RESEARCH*

*BRAIN RESEARCH BULLETIN*

*CLINICAL REHABILITATION*

*EUROPEAN JOURNAL OF NEUROLOGY*

*HEALTH POLICY*

*INTERNATIONAL JOURNAL OF HEALTH SERVICES*

*JOURNAL OF COMORBIDITY*

*JOURNAL OF HEALTH SERVICES RESEARCH AND POLICY*

*THE COCHRANE DATABASE OF SYSTEMATIC REVIEWS*

*THE LANCET. NEUROLOGY*

*and these searches:*

*1 - "integrated care" & "parkinson" & "huntington" & "neurological" & "intersectoral" & "multisectoral" in Title*

*2 - "integrated care" & "long term condition" in Title*

*3 - "integrated care" & "neuro" in Title*

*4 - "coordinated care" & "neuro" in Title*

*5 - "integrated care" & "parkinson" in Title*

*6 - "integrated care" & "huntington" in Title*

*There are now 10 journals and 6 searches in your list*

08/05/2022 + 09/05/2022 – Completed Zetoc alert review archived on Zotero and inbox review (n= 156) – completed. No articles identified (SBP)

**Table S-2.** Alerts created on most relevant Journals

| Journal | Link | Alert | Zetoc |
| --- | --- | --- | --- |
| The Lancet Neurology | <https://www.thelancet.com/journals/laneur/home> | Alert set for Neurology and Public Health, on a weekly basis | Y |
| International Journal of Integrated Care (IJIC) | <https://www.ijic.org/> | Registered and alert set | Cancelled by software |
| BMC Health Services Research | <https://bmchealthservres.biomedcentral.com/> | Alert set | Y |
| Journal of Health Services Research & Policy | <https://journals.sagepub.com/home/hsr> | Alert set |  |
| International journal of health services |  |  | Y |
| Health Policy | <https://www.journals.elsevier.com/health-policy> | Alert set | Y |
| Journal of Comorbidity | <https://journals.sagepub.com/home/cob> | Alert set | Y |
| Cochrane Database of Systematic Reviews |  |  | Y |
| NIHR Prospero |  |  |  |
| Health Services and Delivery Research | <https://www.journalslibrary.nihr.ac.uk/hsdr/#/> |  |  |
| Journal of Parkinson's Disease | <https://www.journalofparkinsonsdisease.com/> | Registered and alert set | Cancelled by software |
| IoS press | <https://content.iospress.com/journals/journal-of-parkinsons-disease/Pre-press/Pre-press> | Registered and saved PD and HD journals. |  |
| Clinical Rehabilitation |  |  | Y |
| European Journal of Neurology |  |  | Y |
| Canadian Journal of Neurological Sciences |  |  | Cancelled by software |
| Brain Research Bulletin |  |  | Y |
| Journal of Huntington's Disease | <https://www.iospress.nl/journal/journal-of-huntingtons-disease/> | Registered and alert set |  |

Hand-searching was performed for publications in the last year before the search happened.

Documenting Hand-searching - Specific titles and date ranges searched for a systematic review should be included in the search strategies section. It should include journal titles, listed in alphabetical order, and the months and years that have been searched. Hand-searching was performed online at the Journals weblink from September 2020 to 12 May2022. Material copies were requested from the University library to cover all numbers within the last year.

**Table S-3.** Hand-searching documentation

| **Journal: The Lancet Neurology, link:** [**https://www.thelancet.com/journals/laneur/home**](https://www.thelancet.com/journals/laneur/home) | | |
| --- | --- | --- |
| Date searched | Names of saved items + reference number | Reviewer initials |
| 09/05/2022 | Zero searches from Sept 2020-Dec2020 | SBP |
| 10/05/2022 | Zero searches from Jan-December 2021 and Jan-May2022 | SBP |
| **Journal: International Journal of Integrated Care (IJIC), link:** [**https://www.ijic.org/**](https://www.ijic.org/) | | |
| Date searched | Names of saved items + reference number | Reviewer initials |
| 10/05/2022 | Zero searches from Sept 2020-Dec2020 | SBP |
| 10/05/2022 | Zero searches from Jan-December 2021 and Jan-May2022 | SBP |
| **Journal: BMC Health Services Research, link:** [**https://bmchealthservres.biomedcentral.com/**](https://bmchealthservres.biomedcentral.com/) | | |
| Date searched | Names of saved items + reference number | Reviewer initials |
| 10/05/2022 | Zero searches from Sept 2020-Dec2020 | SBP |
| 10/05/2022 | Zero searches from Jan-December 2021 and Jan-May2022 | SBP |
| **Journal: Movement disorders: official journal of the Movement Disorder Society, link:** [**https://www.movementdisorders.org/MDS/Journals/Online-MD-Journal.htm**](https://www.movementdisorders.org/MDS/Journals/Online-MD-Journal.htm) | | |
| Date searched | Names of saved items + reference number | Reviewer initials |
| 10/05/2022 | Zero searches from Sept 2020-Dec2020 | SBP |
| 10/05/2022 + 12/05/2022 | Zero searches from Jan-December 2021 and Jan-May2022 | SBP |

For citation searching, Web of Science (access through <https://library.soton.ac.uk/systematic-reviews/health>) was the resource used, after identification of key papers in this review.

**Table S-4.** Citation searching

| Date searched | Original paper (Author, Year) + reference number | Number of citations identified + Papers saved (Author, Year) + reference number | Researcher initials |
| --- | --- | --- | --- |
| 13/05/2022 | Mestre et al 2021 | 3 citations identified. No papers saved. | SBP |
| 13/05/2022 | Kessler et al 2021 | 4 citations identified. 2 papers saved (Grosjean, 2021; Connor 2022) relevant to subject but not matching eligibility criteria. No papers included. | SBP |
| 13/05/2022 | Connor et al 2020 | 4 citations identified. No papers saved. | SBP |
| 13/05/2022 | Connor et al 2019 | 21 citations identified. No papers saved. | SBP |
| 13/05/2022 | Munoz et al, 2020 | 1 citation identified | SBP |
| 13/05/2022 | Fleisher et al, 2020 | 4 citations identified. No papers saved. | SBP |
| 13/05/2022 | Fleisher et al, 2018 | 25 citations identified. No papers saved. | SBP |
| 13/05/2022 | Jones et al, 2016 | 13 citations identified. No papers saved. | SBP |
| 13/05/2022 | van der Marck et al 2013 (The Netherlands) | 78 citations identified. No papers saved. | SBP |
| 13/05/2022 | van der Marck et al 2013 (Canada) | 83 citations identified. No papers saved. | SBP |
| 13/05/2022 | Trend et al 2002 | 80 citations identified. No papers saved. | SBP |
| 13/05/2022 | Healey et al 2019 | 4 citations identified. No papers saved. | SBP |
| 13/05/2022 | Jansen et al 2006 | 2 citations identified. No papers saved. | SBP |
| 13/05/2022 | Oeseburg et al 2004 | 8 citations identified. No papers saved. | SBP |
| 13/05/2022 | Zirra et al 2017 | No results found | SBP |
| 13/05/2022 | Makepeace et al 2001 | 3 citations identified. No papers saved. | SBP |
| 13/05/2022 | Veenhuizen et al 2011 | 26 citations identified. No papers saved. | SBP |
|  |  | Total = 359 |  |

**Table S-5.** Documentation of grey literature search

| Date searched | Source name | Pathway followed, e.g., website browsed/ search engine used (which)/database within website | Predefined keywords:  N (no)  Y (yes)  Add keywords if Y | Screening approach | Papers saved (Author, Year) + reference number | Last date of revision/ saved items | Researcher initials |
| --- | --- | --- | --- | --- | --- | --- | --- |
| 09/05/2022 | Zotero library |  | N | Screened individual papers within literature review | 0 |  | SBP |

# **Appendix 4 Data extraction tool template**

| **Paper Details** | | | | | | | | | | **Inclusion/Exclusion Criteria** | | | | | **Details/results extracted from source of evidence** | | | | | | | | | | | | | **Context of this review** | | | | | Other comments |
| --- | --- | --- | --- | --- | --- | --- | --- | --- | --- | --- | --- | --- | --- | --- | --- | --- | --- | --- | --- | --- | --- | --- | --- | --- | --- | --- | --- | --- | --- | --- | --- | --- | --- |
| Author | Year | Country of origin | Title | Objectives | Questions/Aims | Methodology / Design | Intervention/Programme/Initiative/Model | Intervention intensity | Participants (details e.g., patient/ carer, age/sex and number) | Population (PD, HD, MS) | Study design | Intervention. Concept of integrated care NOT disease management. Multisectoral | Context (settings/sectors involved) | Continuum of care (or specific disease stage) | Integrated care definition and characteristics | Model of care used | Visual representation | PPI | Multidisciplinary, Intersectoral, Multisectoral actions | Roles involved/team composition | Outcomes and details (e.g., how measured, how often, for the patient, caregiver, service, professionals) | Effectiveness assessed, what timepoints | Feasibility details (obstacles and strategies to implementation) | Key Findings/Summary | Future research | Study limitations/Gaps | Funder | Interpretation of findings in the reviewer words | Applicability for this review | Themes | Reviewer initials | Date |  |

# **Appendix 5 Risk of bias assessment**

|  | Study Reference | Study Design | CASP tool used | CASP score | Critical appraisal Observations | Cochrane bias observations |
| --- | --- | --- | --- | --- | --- | --- |
| 1 | Connor et al 2020 | RCT descriptive data | RCT | 6/11 | No analysis between intervention and clinical outcomes.  Lack of understanding on intervention impact per disease stage, no discrimination. No cost-effective analysis.  Samples are quite small and it is not specified on this study how outcomes were assessed. the actual table provided does not match this info, the table specifies participants' concerns, and not improvements. | Due to the nature of the study, staff delivering the intervention and participants were not blinded, this may incur on different types of bias namely performance bias. Potentially threats study validity.  Potential reporting bias due to incoherence in data reported and conclusions reached. |
| 2 | Connor et al 2019 | RCT | RCT | 9/11 | Not ideal blinding  No cost-effectiveness  Baseline PD quality indicator adherence measures were not obtained (the baseline scores would provide information on whether care quality differed at baseline across the usual care and intervention arms) | Due to the nature of the study, staff delivering the intervention and participants were not blinded, this may incur on different types of bias namely performance bias. Potentially threats study validity. |
| 3 | van der Marck et al 2013 (Canada) | RCT | RCT | 9/11 | No analysis on the dropouts, unable to perform intention-to-treat analysis.  The study excluded more advances pts, potentially that could have received most benefit of the intervention. | Patients were not blinded due to the nature of intervention, this may incur in performance bias. |
| 4 | van der Marck et al 2013 (The Netherlands) | non-RCT | RCT | 7/11 | No randomization.  Study excluded the more advanced and vulnerable patients, potentially excluding the pts who could have benefited more from this integrated care approach.  Research staff was not blinded, clinicians and PD nurses had some awareness of the study but did not know which patients had been recruited. | Possible selection bias due no non-randomization. Not all staff involved was blinded. |
| 5 | Kessler et al 2021 | Qualitative | Qualitative | 9/10 | Recruitment strategy could have been more representative of participants. | Selection bias due to choosing participants and have HCP on focus groups that were also researchers. |
| 6 | Oeseburg et al 2004 | Open case-study | Cohort | 8/14 | Recruitment is not clear and no drop-out info is given (just the number of patients decreased from 40 to 38)  Analysis – no confidence intervals provided  Not enough info about the researchers and clinicians, was it the same team providing and analysing care? | Possible selection bias due to unclear recruitment process.  Possible performance bias due to researchers/clinicians’ logistics, which is unclear. |
| 7 | Zirra et al 2017 | Prospective | Cohort | 9/14 | Recruitment is not clear. No blinding, clinicians and researchers were the same and questionnaires were not anonymous. No limitations in the study considered. | Possible performance bias and detection bias since clinical team/researchers administered the outcome scale directly to patient. Subjects and outcome assessors were not blinded to exposure. |
| 8 | Makepeace et al 2001 | Prospective | Cohort | 11/14 | There seems to be no blinding, no information on who/how administered the scales to measure patient outcomes.  There is no data available to assess satisfaction before and after the intervention.  Outcomes chosen may not be the best to meet the study objective.  Study lacks methodological depth. | Possible performance bias and detection bias since clinical team/researchers administered the outcome scale directly to patient. Subjects and outcome assessors were not blinded to exposure. |
| 9 | Fleisher et al 2018 | Retrospective | Cohort | 12/14 | There seems to be no blinding.  Follow-up/data collection could have been more complete with data missing e.g., fall rates, hospital days, and costs.  Low response rate to questionnaire - social desirability bias.  Data assessed but not reported e.g., caregivers strain index | Possible performance bias and detection bias since clinical team/researchers administered the outcome scale directly to patient. Subjects and outcome assessors were not blinded to exposure.  Attrition bias due to lack of information on loss to follow-up  Reporting bias due to data being assessed but not reported e.g., caregivers strain index. |
| 10 | Fleisher et al 2020 | Before-after | Cohort | 13/14 | There seems to be no blinding between patients/outcome assessors and exposure to intervention.  Most vulnerable patients excluded from intervention limiting generalizability. No costs data. No controls on this intervention. | Possible performance bias and detection bias since clinical team/researchers administered the outcome scale directly to patient. Subjects and outcome assessors were not blinded to exposure. |
| 11 | Mestre et al 2021 | Before-after | Cohort | 12/14 | There seems to be no blinding between patients/outcome assessors and exposure to intervention.  Setting very different from local setting – harder to translate evidence | Possible performance bias and detection bias since clinical team/researchers administered the outcome scale directly to patient. Subjects and outcome assessors were not blinded to exposure. |
| 12 | Trend et al 2002 | Before-after | Cohort | 13/14 | There seems to be no blinding between patients/outcome assessors and exposure to intervention. No control group. | Possible performance bias and detection bias since clinical team/researchers administered the outcome scale directly to patient. Subjects and outcome assessors were not blinded to exposure. |
| 13 | Healey et al 2019 | Before-after | Cohort | 11/14 | There seems to be no blinding between patients/outcome assessors and exposure to intervention. No control group.  Very small sample.  Outcomes assessed very limited and not validated. E.g., EDSS scores baseline/follow-up missing | Possible performance bias and detection bias since clinical team/researchers administered the outcome scale directly to patient. Subjects and outcome assessors were not blinded to exposure.  Selection bias (pointed out by authors)  Reporting bias due to some outcomes being assessed but not reported. |
| 14 | Jansen et al 2006 | Quantitative | Cohort | 9/14 | There seems to be no blinding between patients/outcome assessors and exposure to intervention.  Differences between baseline control and intervention groups.  Absence of information concerning persons who declined to participate or dropped out of the study and outcomes such as care continuity.  Some outcomes not validated for this population. | Possible performance bias and detection bias. Subjects were not blinded to exposure.  Selection bias due to differences between control and intervention groups.  Reporting bias due to missing information on people who declined participation and withdrawn from the study.  Detection bias due to some measurement scales not validated for this specific population. |
| 15 | Munoz et al 2020 | Post-intervention survey | Cohort | 5/14 | There seems to be no blinding between patients/outcome assessors and exposure to intervention.  HCP were participants as well.  Unclear how long was each dyad exposed to intervention at assessment time.  No control, no baseline data.  No info on patient retention through intervention  QoL and burden mentioned but not reported.  Flawed design and methods | Possible performance bias and detection bias. Subjects were not blinded to exposure.  Potential performance bias since unclarity in how long each dyad was exposed to intervention at assessment time.  Detection bias since it seems to be no blinding between patients/outcome assessors and exposure to intervention. Unclear how HCP were blinded to the intervention or how much they were involved in delivering the intervention.  Reporting bias due to some outcomes mentioned but not reported. |

# **Appendix 6 Outcomes table of included studies**

**Table S-6.** Outcomes and scales of the included studies for patients and caregivers

| Study ID | Parkinson’s Disease Questionnaire–8 (PDQ-8) | Movement Disorder Society–Unified Parkinson’s Disease Rating Scale (MDS-UPDRS) Part I – non-motor | Movement Disorder Society–Unified Parkinson’s Disease Rating Scale (MDS-UPDRS) Part II - motor | Movement Disorder Society–Unified Parkinson’s Disease Rating Scale (MDS-UPDRS) Part III – motor examination physician | Movement Disorder Society–Unified Parkinson’s Disease Rating Scale (MDS-UPDRS) Part IV – motor complications | Movement Disorder Society–Unified Parkinson’s Disease Rating Scale (MDS-UPDRS) TOTAL score | Hoehn and Yahr | Emerson and Enderby measures of voice and articulation | Gait – timed walk over 10 metres | EDSS Kurtzke’s Expanded Disability Status Scale | Incapacity Status Scale (ISS) | Environmental Status Scale (ESS) | Functional Independence Measure (FIM.) | Mini mental state examination | Unified Parkinson’ s Disease Rating  Scale Activities of Daily Living | Act daily living Academic Medical Center linear disability score (ALDS) | Geriatric Depression Scale | Depression (Montgomery-Asberg Depression Scale (MADRS) | Hospital Anxiety and Depression Scale (HAD) patient | Newcastle Independence Assessment Form (NIAF) | Clinical Global Impression–Change and Clinical Global Impression–Satisfaction Scales  physician | Patient Assessment of Chronic Illness Case+ (PACIC+) | Self-management support 5As (within PACIC+) | PD Quality indicator Communication, education & continuity | PD Quality indicator Health maintenance | PD Quality indicator Regulatory reporting | PD Quality indicator assessment of nonmotor symptoms and falls | PD Quality indicator assessment and counselling about PD medication side effects | PD Quality indicator management of motor symptoms | PD Quality indicator management of nonmotor complications of PD | PD Quality indicator use of nonpharmacologic approaches and therapies | PD Quality indicator Palliative care | Health Utilities Index 3 | Consumer Assessment of Healthcare Providers and Systems [CAHPS] | Medical Outcomes Study [MOS] Social Support Scale | General Self-Efficacy Scale | Psychosocial functioning (Scales for Outcomes in Parkinson’s disease-Psychosocial (SCOPA-PS) | WHO Well-being Index–5 | Neuro-QoL | Parkinson’s disease quality of life questionnaire (PDQL]) | Parkinson’s disease QoL questionnaire (PDQ-39) | Euroqol-5d (EQ-5d) patient | Health-related QOL (RAND-36) | Health state utility values (HSUVs) | General Health Questionnaire (GHQ) | Patient Healthcare Questionnaire [PHQ]–2 | Patient Healthcare Questionnaire PHQ-9 | Healthcare use questionnaire | Healthcare needs adapted from International Classification of Impairments, Disabilities, and Handicaps (ICIDH) | Continuity of care (Vragenlijst Continuïteit van Zorg) | Client Satisfaction Inventory-Short Form (CSI-SF) patient | Satisfaction with programme Likert scale patient | Satisfaction with programme Likert scale caregiver | Client Satisfaction Inventory-Short Form (CSI-SF) caregiver | Multidimensional Caregiver Strain Index (MCSI) |  | Zarit Caregiver Burden Questionnaire | Caregiver burden belastungsfragebogen Parkinson angehörigen–kurzversion (BELA-A-k) | Caregiver Strain Index (CSI) | Hospital Anxiety and Depression Scale (HAD) caregiver | Euroqol-5d (EQ-5d) caregiver |
| --- | --- | --- | --- | --- | --- | --- | --- | --- | --- | --- | --- | --- | --- | --- | --- | --- | --- | --- | --- | --- | --- | --- | --- | --- | --- | --- | --- | --- | --- | --- | --- | --- | --- | --- | --- | --- | --- | --- | --- | --- | --- | --- | --- | --- | --- | --- | --- | --- | --- | --- | --- | --- | --- | --- | --- | --- | --- | --- | --- | --- | --- |
| Mestre et al 2021 (0-6M) | O↑ | O↔ | O↔ | O ↑ |  |  |  |  |  |  |  |  |  |  |  |  | O? |  |  |  | O? | O↑ | O↑ |  |  |  |  |  |  |  |  |  |  |  |  |  |  |  |  |  |  |  |  |  |  |  |  |  |  |  |  | O  Care priorities met 90.6% cases  Satisfaction very high 77% |  |  |  |  | O↔ |  |  |  |  |
| Kessler et al 2021 |  |  |  |  |  |  |  |  |  |  |  |  |  |  |  |  |  |  |  |  |  |  |  |  |  |  |  |  |  |  |  |  |  |  |  |  |  |  |  |  |  |  |  |  |  |  |  |  |  |  |  | Q Coordinator support  New knowledge  Goal Identification and achievement  Self-management |  |  |  |  |  |  |  |  |  |
| Connor et al 2020 |  |  |  |  |  |  |  |  |  |  |  |  |  |  |  |  |  |  |  |  |  |  |  |  |  |  |  |  |  |  |  |  |  |  |  |  |  |  |  |  |  |  |  |  |  |  |  |  |  |  |  | Q Patients most frequently reported improvements were: Motor-related, Sleep and fatigue, Weight/nutrition/dental, Pain, Swallowing, Depressive symptomatology, and Falls |  |  |  |  |  |  |  |  |  |
| Connor et al 2019 (0-18M/24M) |  |  |  |  |  |  |  |  |  |  |  |  |  |  | X↔ |  |  |  |  |  |  | O↔ |  | O↑ | O↔ | O↔ | O↑ | O↑ | O↑ | O↑ | O↑ | O↑ | X↔ | X↔ | X↔ | X↔ |  | X↔ |  |  |  |  |  |  |  | X↑ | X↔ |  |  |  |  | 70% reported their nurse care manager helped them to manage their PD and 77% reported their NCM helped them to be safe and active |  |  |  |  |  |  |  |  |  |
| Fleisher et al 2020 (0-12M) |  | O↑ | O↔ | O↑ | O↔ | O↑ | O↔ |  |  |  |  |  |  | O↔ |  |  |  |  |  |  |  |  |  |  |  |  |  |  |  |  |  |  |  |  |  |  |  |  | O↔ |  |  |  |  |  |  |  |  |  |  |  | Score 97 |  |  | Score 98 | O↑ |  |  |  |  |  |  |
| Fleisher et al 2018 |  |  |  |  |  |  |  |  |  |  |  |  |  |  |  |  |  |  |  |  |  |  |  |  |  |  |  |  |  |  |  |  |  |  |  |  |  |  |  |  |  |  |  |  |  |  |  |  |  |  | Score 96 |  |  | Score 98 |  |  |  |  |  |  |  |
| Munoz et al 2020 (0-24M) |  |  |  |  |  |  |  |  |  |  |  |  |  |  |  |  |  |  |  |  |  |  |  |  |  |  |  |  |  |  |  |  |  |  |  |  |  |  |  |  | O? |  |  |  |  |  |  |  |  |  |  | 89.5% rated the activities as excellent. 87.9% considered that the activities have improved their QoL. |  |  |  |  | O? |  |  |  |  |
| van der Marck et al 2013 (0-8M) Nether |  |  |  | X↔ |  |  |  |  |  |  |  |  |  |  |  | O↔ |  |  |  |  |  |  |  |  |  |  |  |  |  |  |  |  |  |  |  |  |  |  |  | O↔ |  |  |  |  |  |  |  |  |  |  |  |  |  |  |  |  |  | X↔ |  |  |  |
| van der Marck et al 2013 (0-8M) Canad |  |  |  | X ↑ |  | X ↑ |  |  |  |  |  |  |  |  |  |  |  | X ↑ |  |  |  |  |  |  |  |  |  |  |  |  |  |  |  |  |  |  | X ↑ |  |  |  | O ↑ mobility, emotional |  |  |  |  |  |  |  |  |  |  |  |  |  |  |  |  |  | X↔ |  |  |
| Trend et al 2002 (0-6weeks) |  |  |  |  |  |  |  | O↑ | O↑ |  |  |  |  |  |  |  |  |  | O↑ |  |  |  |  |  |  |  |  |  |  |  |  |  |  |  |  |  |  |  |  |  |  | O↑ |  |  |  |  |  |  |  |  |  | Knowledge PD increase pts+carers |  |  |  |  |  |  |  | O↔ | O↔ |
| Healey et al 2019 (-12M-0-12M) |  |  |  |  |  |  |  |  |  |  |  |  |  |  |  |  |  |  |  |  |  |  |  |  |  |  |  |  |  |  |  |  |  |  |  |  |  |  |  |  |  |  |  |  |  |  |  |  |  |  |  | Average pt satisfaction 30/32 |  |  |  |  |  |  |  |  |  |
| Zirra et al 2017 (0-8weeks) |  |  |  |  |  |  |  |  |  |  |  |  |  |  |  |  |  |  |  |  |  |  |  |  |  |  |  |  |  |  |  |  |  |  |  |  |  |  |  |  |  | O↑  Mobility, activities, pain |  | 81%improved |  |  |  |  |  |  |  |  |  |  |  |  |  |  |  |  |  |
| Jansen et al 2006 (0-10M) |  |  |  |  |  |  |  |  |  |  |  |  |  |  |  |  |  |  |  |  |  |  |  |  |  |  |  |  |  |  |  |  |  |  |  |  |  |  |  |  |  |  | O↑ Energy, less ∆in health |  |  |  |  | O↑GP, neurologist etc | O↑ personal care, defecation & vision | O↔ |  |  |  |  |  |  |  |  |  |  |  |
| Oeseburg et al 2004 (0-15M) |  |  |  |  |  |  |  |  |  | O↔ | O↔ | O↔ |  |  |  |  |  |  |  |  |  |  |  |  |  |  |  |  |  |  |  |  |  |  |  |  |  |  |  |  |  |  | O↔ |  |  |  |  |  |  |  |  | 2/3 needs met  Expressed needs decreased |  |  |  |  |  |  |  |  |  |
| Makepeace et al 2001 (0-6M) |  |  |  |  |  |  |  |  |  | O↓ |  |  | O↓ |  |  |  |  |  |  | O↔ |  |  |  |  |  |  |  |  |  |  |  |  |  |  |  |  |  |  |  |  |  |  |  |  | O↔ |  |  |  |  |  |  | very satisfied score 26.5/30 accessibility, Home Visits, continuity etc |  |  |  |  |  |  |  |  |  |

O = Primary outcome

X = Secondary outcome

Q = Qualitative analysis

↑ = statistically significant improvement in outcome following intervention (p≤0.05)

↓ = statistically significant decline in outcome following intervention (p≤0.05)

= not statistically significant improvement in outcome following intervention (p>0.05)

? = not reported

∆ = change

PDQ-8, Parkinson’s Disease Questionnaire–8;

MDS-UPDRS, Movement Disorder Society–Unified Parkinson’s Disease Rating Scale;

PACIC, Patient Assessment of Chronic Illness Case that measures the specific actions or qualities of care that patients report they have experienced in the delivery system congruent with the CCM (PACIC) and more specifically concerning self-management support (5As)

**Table S-7.** Outcomes related to service impact on included studies

| **Study ID** | **A&E visits** | **Hospitalizations** | **Length of stay Hospital** | **Nursing home admissions** | **LoS Nursing home** |
| --- | --- | --- | --- | --- | --- |
| Fleisher et al 2018 | O↓ | O↓ |  |  |  |
| Healey et al 2019 | O↔ | O↔ | O↔ | O↓ | O↓ |
| Makepeace et al 2001 |  |  | O↓ |  |  |

↓ = reduced compared to standard of care and/or baseline

↔ = no difference compared to standard of care and/or baseline

# **Appendix 7 References**

1. MIMAS. Zetoc Homepage. Accessed November 22, 2020. https://zetoc.jisc.ac.uk/

2. Liberati A, Altman DG, Tetzlaff J, et al. The PRISMA Statement for Reporting Systematic Reviews and Meta-Analyses of Studies That Evaluate Health Care Interventions: Explanation and Elaboration. *PLOS Med*. 2009;6(7):e1000100. doi:10.1371/journal.pmed.1000100
